# Dietary tryptophan deficiency promotes gut RORγt^+^ Treg cells at the expense of Gata3^+^ Treg cells and alters commensal microbiota metabolism

**DOI:** 10.1016/j.celrep.2023.112135

**Published:** 2023-02-24

**Authors:** Lucille C. Rankin, Katherine A. Kaiser, Kenia de los Santos-Alexis, Heekuk Park, Uhlemann Anne-Catrin, Daniel H.D. Gray, Nicholas Arpaia

**Affiliations:** 1 Department of Microbiology & Immunology, Columbia University Irving Medical Center, 701 West 168th Street, HHSC 910C, New York, NY, USA; 2 Microbiome & Pathogen Genomics Core, Columbia University Irving Medical Center, New York, NY, USA; 3 Division of Infectious Diseases, Department of Medicine, Columbia University Irving Medical Center, New York, NY, USA; 4 Walter and Eliza Hall Institute of Medical Research, Parkville, VIC, Australia; 5 Department of Medical Biology, University of Melbourne, Parkville, VIC, Australia; 6 Lead contact

## Abstract

Micronutrient deficiency is a major cause of disease throughout the world. Yet, how perturbations influence the immune-microbiome interface remains poorly understood. Here, we report that loss of dietary tryptophan (Trp) reshapes intestinal microbial communities, including the depletion of probiotic *L. reuteri*, drives transcriptional changes to immune response genes in the intestinal ileum, and reshapes the regulatory T cell (Treg) compartment. Dietary Trp deficiency promotes expansion of RORγt^+^ Treg cells and the loss of Gata3^+^ to Tregs in a microbiota-dependent manner. In the absence of dietary Trp, provision of the AhR ligand indole-3-carbinol is sufficient restore the Treg compartment. Together, these data show that dietary Trp deficiency perturbs the interaction between the host and its bacterial symbionts to regulate Treg homeostasis via the deprivation of bacterially derived Trp metabolites. Our findings highlight an essential role for immune-microbiome crosstalk as a key homeostatic regulator during nutrient deficiency.

## In brief

Rankin et al. show that dietary tryptophan deficiency reshapes the microbiota and alters intestinal gene expression profiles, resulting in a microbiota-dependent expansion of gut RORγt^+^ Tregs. These findings highlight the potential for harnessing dietary immune modifiers, such as tryptophan, to regulate RORγt^+^ Treg numbers and possibly treat intestinal inflammatory disorders.

## Graphical Abstract

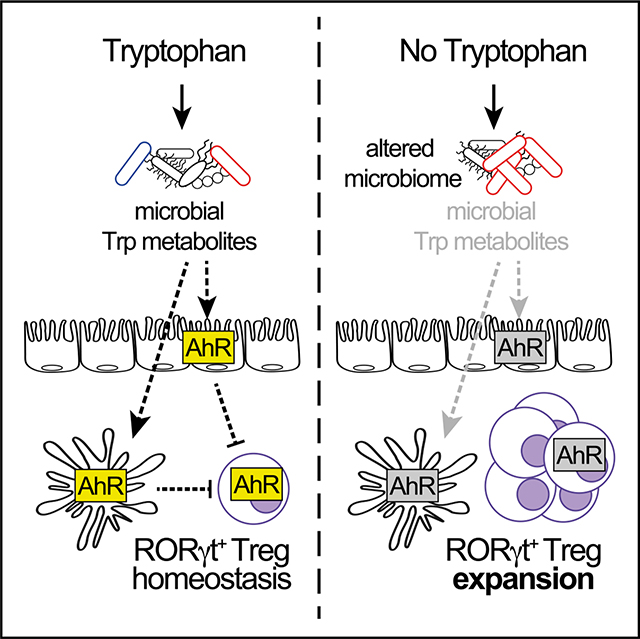

## INTRODUCTION

Human and mammalian diets vary greatly across geographical areas, cultural groups, and age. Throughout life, nutrient consumption can fluctuate widely between periods of abundance and scarcity. Chronic malnutrition is a major cause of morbidity and mortality throughout the world that leads to illnesses related to immune function, gastrointestinal (GI) health, and metabolism. The GI tract is a multi-organismal ecosystem that facilitates the absorption of nutrients across these diverse conditions. In particular, the small intestine (SI) is uniquely adapted to both sense and absorb nutrients from diverse food sources and protect the host from toxic and pathogenic onslaughts. It is also appreciated that intestinal microbes have co-evolved to regulate host physiology, metabolism, and immunity through the biosynthesis and provision of vitamins and essential amino acids, fermentation of carbohydrates into bioactive short chain fatty acids (SCFA), and the biotransformation of bile acids.^1–18^ We therefore hypothesized that during acute nutrient deficiency, immunosuppressive regulatory T cells (Tregs) may also coordinate and respond to changes in nutrient availability in the SI to regulate host homeostasis.

In the GI tract, FoxP3^+^ regulatory T cells are a unique subset of immunosuppressive CD4^+^ T cells that promote tolerance to food antigens and gut microbes. The mammalian gut contains two dominant populations of Treg cells defined by their expression of the transcription factors Gata3 and retinoid-related orphan receptor-γt (RORγt). RORγt^+^ Treg cells are generated in the periphery from naive CD4^+^ T cells in response to factors derived from diet and commensal bacteria, such as retinoic acid, secondary bile acids, and SCFAs that make up the enteric microenvironment.^19–21^ In contrast, Gata3^+^ Tregs arise in the thymus, develop central tolerance to self-antigens and are acutely responsive to the alarmin IL-33.^19,21–26^ We therefore hypothesized that removal of an essential dietary nutrient would fundamentally alter the microbial ecosystem and thus the repertoire of bacterial metabolites that regulate intestinal Treg cell homeostasis.

We developed a mouse model of acute dietary tryptophan (Trp) deficiency to study metabolite-based communication between the diet, commensal microbial community, and intestinal Tregs. Trp is an essential amino acid that cannot be synthesized *de novo* by host metabolic pathways. Dietary Trp is absorbed primarily in the SI and is essential for many aspects of host physiology.^27–31^ At least 90% of Trp is metabolized by host indoleamine 2,3-dioxygneases (IDO) to form kynurenines, and the remainder is subject to metabolism by Trp hydroxylases (TPH1 and TPH2), into the neurotransmitter serotonin. Intestinal commensal bacteria are proficient in the biosynthesis and utilization of Trp as an energy source, catabolizing Trp into a wide array of indole-containing molecules that activate AhR-mediated transcription to affect host metabolism, mucosal barrier integrity, and immune responses.^17,32–37^

Here we report that acute dietary Trp deficiency significantly alters the intestinal microbiota and gut metabolites, which in turn drive transcriptional changes in immune response genes in ileal tissue. In particular, the composition of GI Treg cell populations is remodeled by acute dietary Trp deficiency; absence of dietary Trp drives the proliferative expansion of Foxp3^+^RORγt^+^Treg cells and concomitantly suppresses the generation of Gata3^+^ Treg cells in a microbiota-dependent manner. Provision of the potent AhR ligand, indole-3-carbinal (I3C), is sufficient to block the expansion of RORγt^+^ Treg cells induced by a Trp-deficient diet. Therefore, bacterial metabolism of dietary Trp into AhR ligands acts within the intestinal mucosa to inhibit the expansion of RORγt^+^ Treg cells and maintain a balance with Gata3^+^ Treg cells. This study demonstrates a mechanism that shapes host-commensal mutualism during periods of nutrient deficiency and informs how dietary interventions might direct the mucosal immune response to treat intestinal inflammatory disease.

## RESULTS

### Acute Trp deficiency alters microbiota composition and depletes critical Trp metabolites

**Figure 1.**
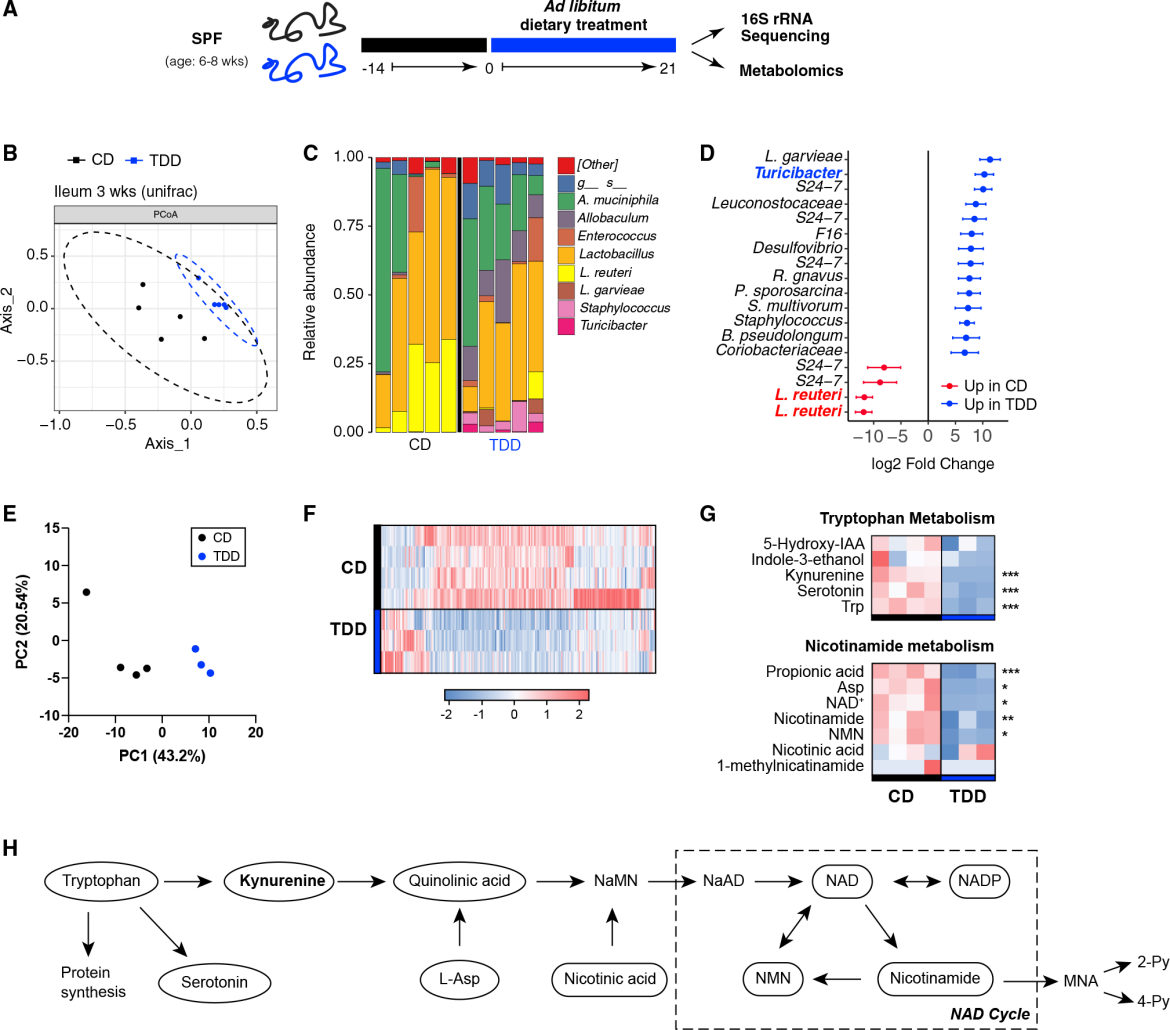
A Trp-deficient diet changes the ileal microbiota and fecal metabolome SPF mice were fed Bakers Amino Acid diet (CD) for 1–2 weeks, then placed on Bakers Amino Acid diet without Trp (TDD) for 3 weeks. (A) Ileal contents and/or feces were collected followed by 16S rRNA sequencing and CE-TOFMS metabolite analysis. (B) A Bray-Curtis PCoA plot representing the differences in the beta diversity of bacterial communities in the ileum of SPF mice fed CD or TDD. (C) Relative abundance of main bacterial genus detected within the ileum of SPF mice fed CD or TDD at 3 weeks. (D) Forest plot that shows Log_2_FC of the most differentially abundant taxa within the ileum of SPF mice fed CD compared with those fed TDD. Data are representative of two experiments (n = 4–5 mice per group/experiment); error bars represent SD. (E) Unbiased metabolite analysis was carried out in the stool of animals fed CD or TDD using CE-TOFMS (E–H) and principal-component analysis of detected metabolites is shown in (E). (F) Differentially abundant known putative metabolites were detected and shown as a heatmap in (F). (G) Heatmaps of selected metabolites identified in the stool of SPF mice fed CD or TDD that are involved in tryptophan metabolism (upper) and nicotinamide metabolism (lower). (F) is a schematic representation of pathways of Trp metabolism. p values in (F) and (G) were computed by Welch’s t test. (*<0.05, ^**^<0.01, ^***^<0.001). Heatmaps were generated using standardized value of relative area in detected peaks. Data are from one independent experiment (n = 3–4 mice/group). CD, control diet; CE-TOFMS, capillary electrophoresis time-of-flight mass spectrometry; TDD, Trp-deficient diet. See also (Figure S1).

To understand the influence of Trp deficiency on intestinal immune homeostasis, we first assessed whether the composition of intestinal microbiota communities and their metabolic output were modulated by the absence of dietary Trp. Mice were fed control (CD) or Trp-deficient diets (TDD) (Table 1) for 3 weeks and the microbial consortia present in the stool and ileum was determined by 16S rRNA gene sequencing (Figure 1A). First, dramatic weight loss was observed in mice fed a TDD (Figure S1A). This finding is consistent with several reports examining the impact of dietary Trp deficiency in mice and rats.^38,39^ Further, principal-coordinate analysis (PCoA) revealed a significant re-organization of the intestinal microbiota in response to dietary Trp deficiency (Figures 1B and S1B). More specifically, there was an outgrowth of *Turicibacter* spp, and *L. garvieae*, in the absence of dietary Trp; however, no apparent selection for Trp-producing bacteria that may compensate for the loss of dietary Trp was observed (Figures 1C and 1D). The probiotic *L. reuteri* was severely depleted in the absence of dietary Trp (Figures 1C and 1D), which was further confirmed by qPCR analysis (Figure S1C). Similar results were observed in the stool of animals fed TDD for 2, 3, and 5 weeks (Figures S1B, S1D, and S1E). *L. reuteri* uses L-Trp to produce indole derivatives such as indole-3-lactic acid (ILA) that act as AhR ligands that modulate many immune and physiological responses.^23,32,40^ These data together suggest that removal of dietary Trp shifts the microbial ecosystem to disadvantage species such as *L. reuteri*, which use L-Trp as a metabolic substrate.

Since microbial communities were strongly modified after dietary Trp deficiency, we next examined the impact of dietary Trp deficiency on fecal metabolites. We performed untargeted metabolic profiling by capillary electrophoresis time-of-flight mass spectrometry (CE-TOFMS) and found many metabolites were significantly altered in abundance between control and TDD-fed mice (Figures 1E–1G). As expected, Trp itself was among the most strongly depleted metabolites, along with host-derived Trp catabolites serotonin, kynurenine, and nicotinamide (Figures 1G and 1H). Several metabolites involved in purine and pyrimidine biosynthesis were also depleted, as were those involved in cyanoamino acid metabolism, cysteine metabolism, histidine metabolism, and lysine biosynthesis pathways (Figures S1F–S1H). Together, these data demonstrate that dietary Trp deficiency leads to a metabolic shift within the GI tract. Further, our data suggest that the microbiome cannot elevate Trp biosynthesis to compensate for loss of dietary Trp.

### The gut microbiota drives ileal transcriptional responses to dietary Trp deficiency

**Figure 2.**
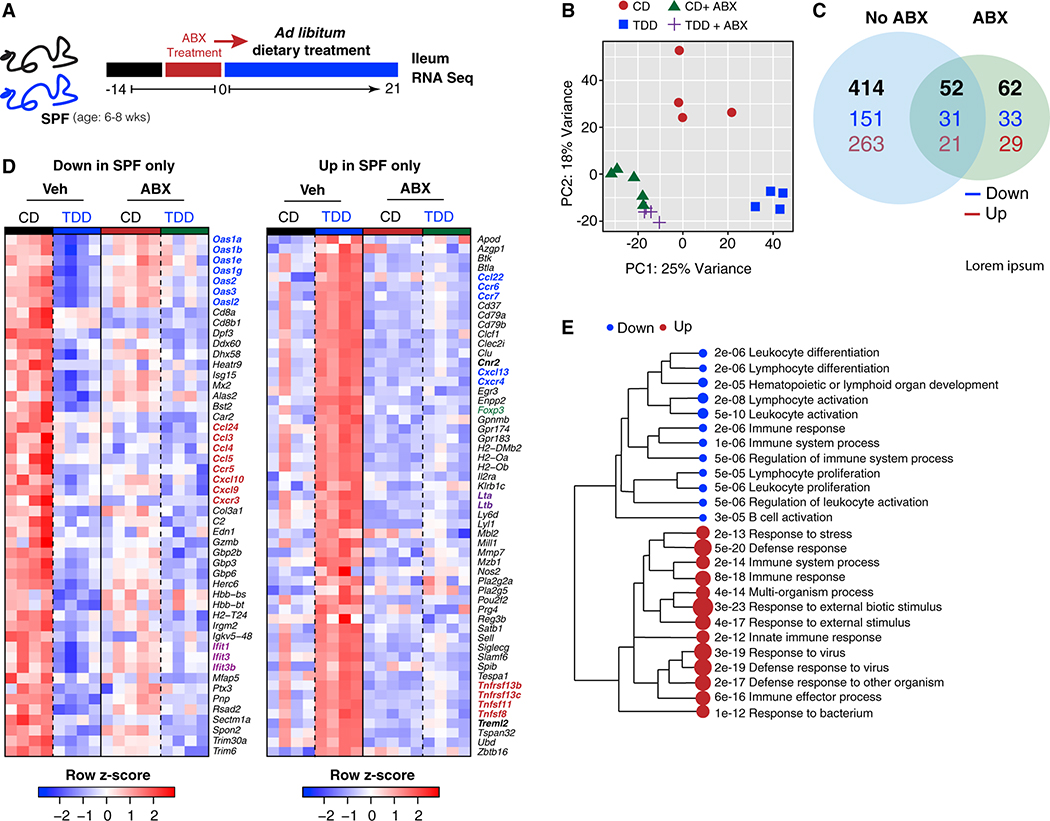
Dietary Trp deficiency profoundly alters ileal gene expression networks in a microbiota-dependent manner (A–C) SPF and antibiotics (ABX)-treated mice were fed CD or TDD for 3 weeks and transcriptional analysis was performed on the terminal ileum (A). Shown in (B) is a principal-component analysis (PCA) plot of gene expression within the ileum of SPF or ABX-treated mice fed a CD or TDD. Lists of differentially expressed genes were generated between groups (q < 0.05) and shown in (C) as a Venn diagram representing the intersection of differentially expressed genes in SPF mice fed CD versus TDD and ABX-treated mice fed CD versus TDD (black is total number of differentially expressed genes, blue is downregulated genes, and red is upregulated genes). GO term enrichment analysis of biological process was carried out on differentially expressed genes between CD versus TDD. (D) Heatmap of downregulated genes (left) and upregulated genes (right) within the GO term immune system process CD versus TDD. (E) Statistically significant GO term enrichment analysis of biological process carried out using the list of differentially regulated genes in SPF mice only. Respective dot size and numbers represent relative and actual p values. Data are from one experiment, n = 4–5 mice per group. ABX, pre-treated with antibiotics (ampicillin, gentamycin, vancomycin, metronidazole, and neomycin) to deplete microbiota; CD, control diet; SPF, specific pathogen free; TDD, Trp-deficient diet. See also Figure S2.

Given the microbial and metabolic changes observed in the GI tract of TDD-fed mice, we next explored how Trp deficiency modified gene expression in the terminal ileum in the presence and absence of an intact microbiome. RNA sequencing (RNA-seq) analysis was performed on the terminal ileum of CD- and TDD-fed mice that remained specific pathogen-free (SPF) or were treated with a cocktail of antibiotics (ampicillin, vancomycin, metronidazole, neomycin, and gentamycin) (ABX) to remove the microbiota (Figure 2A). First, weight loss of ABX-treated animals fed a TDD was much less severe than SPF mice fed a TDD, suggesting the microbiome was exacerbating the observed weight loss (Figure S2A). Second, and most remarkably, we observed a large shift in gene expression in the ileum after TDD feeding that was severely curtailed in ABX-treated animals (Figure 2B). TDD feeding modified the expression of 414 genes in SPF mice and only 114 genes in ABX-treated mice, of which 52 were shared with SPF animals (Figure 2C). This finding suggested that dietary Trp deficiency did not directly drive most of the broad changes in ileal gene expression patterns; rather, most gene expression changes observed in ileal tissue in response to dietary Trp deficiency required the microbiota.

Genes modified by dietary Trp depletion alone included those involved in host Trp metabolism, such as Ido, which encodes *Ido*, the rate-limiting enzyme required for metabolism of Trp into kynurenines (Figures S2B and S2C).^41^ As expected, Trp deficiency significantly altered the expression patterns of the circadian rhythm genes *Nfil3*, *Arntl*, *Bhle41*, and *Per3* (Figures S2B and S2C), given that the circadian rhythm hormones, melatonin and serotonin, are derivatives of host Trp metabolism.^42–44^

As noted above, most genes modified by dietary Trp deficiency required a normal microbiota. Our first consideration was that loss of dietary Trp could reduce barrier integrity, allowing for bacterial translocation.^45^ Therefore, we took a closer look at the pathways modulated by TDD feeding in the intestinal ileum. Most notably, the microbiota was required to induce modifications in immune response gene networks driven by dietary Trp deficiency, but epithelial barrier integrity genes did not appear to be modulated (Figures 2D, 2E, and S2B–S2F). TDD diet fed SPF mice showed decreased expression of numerous CC and CXC chemokines and chemokine receptors (Ccl3,4,5,24 and Ccr5; Cxcl9,10 and Cxcr3), and interferon response genes, including members of the 2–5 oligoadenylate synthetase (OAS) family of genes and interferon-induced proteins with tetratricopeptide repeats (IFITs) (Figure 2D). If there was significant bacterial translocation, these genetic pathways would be expected to be upregulated, suggesting these changes were not in response to inflammation. Those immune response genes induced by dietary Trp deficiency included CX chemokines and chemokine receptors *Cxcl13* and *Cxcr4*, *Foxp3*, *Il2ra*, *Lta*, *Ltb*, and various TNF receptors (Figures 2D, 2E, S2D, and S2E). Dietary Trp deficiency resulted in the induction of several anti-inflammatory genes, including FoxP3 and IL2Ra, providing further evidence that TDD-fed mice were not responding to bacterial translocation. Finally, we investigated those differentially expressed genes not involved in the immune response, which included those involved in amino acid metabolism and biosynthesis, arachidonic acid metabolic processes, and solute carrier (SLC) transporters (Figures S2E and S2F). SLC transporters are responsible for shuttling amino acids and nucleotides across cell membranes and may be upregulated to compensate for nutrient depletion during TDD feeding. In summary, dietary Trp deficiency acts via a complex microbiota to modify the immune compartment of the intestinal ileum.

### Tryptophan deficiency increases RORγt^+^ Treg cells and reduces Gata3^+^ Treg cells

**Figure 3.**
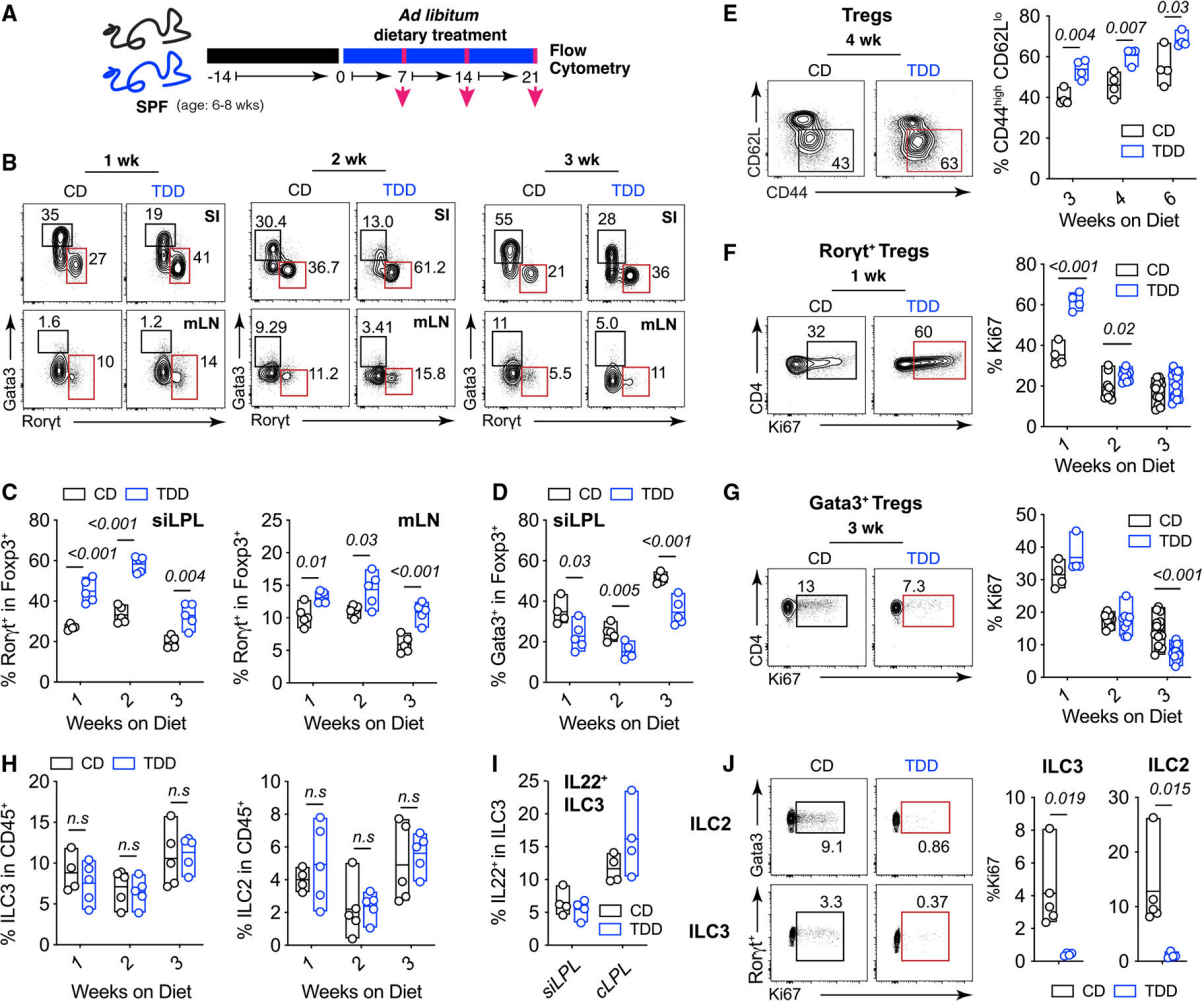
Dietary Trp deficiency drives expansion of intestinal RORγt^+^ Treg cells and loss of Gata3^+^ Treg cells (A) SPF mice were fed CD for 2 weeks, and switched to TDD for 1, 2, or 3 weeks. (B) Representative FACs plots and frequencies of Treg populations (gated on TCRb^+^NK1.1^−^CD4^+^Foxp3^+^ cells) in the indicated organs after 1, 2, and 3 weeks on a CD or TDD diet. (C and D) Quantification of mean (±min/max) frequencies of RORγt^+^ (C) and Gata3^+^ (D) Treg cells isolated from the siLPL and mLN of mice fed control (black) or Trp-deficient diets (blue) for the indicated timepoints. Data are representative of at least two independent experiments (n = 3–5 mice/group). (E) A representative FACs plot of CD44 versus CD62L expression within Tregs isolated from the mLN at the indicated timepoints after TDD feeding. (F and G) Representative FACs plots (left) and frequencies (right) of Ki67-positive cells within RORγt^+^FoxP3^+^ T cells (F) and Gata3^+^FoxP3^+^ T cells (G) at the indicated timepoints. Data are representative of at least two independent experiments where n = 3–5 mice per group. Weeks 2 and 3 in (F) and (G) are pooled from two independent experiments. (H–J) Numbers and function of ILC populations isolated from CD- and TDD-fed mice. Frequency of ILC3 (RORγt^+^ Lin^Neg^) (left) and ILC2 (Gata3^+^ Lin^Neg^) (right) within CD45^+^ si-LP lymphocytes are shown in (H). (I) is frequency of IL22^+^ ILC3 isolated from the si-LP after 3 weeks on CD or TDD and re-stimulated with PMA/ionomycin and IL-23 for 3 h. Representative plots of Ki67^+^ ILC2 (top) and Ki67^+^ ILC3 (bottom) isolated from the siLPL of CD- and TDD-fed mice are shown in (I). Frequencies are enumerated on the right. Data are representative of at least three independent experiments, where n = 3–5 mice per group. Statistical analysis was performed using two-tailed Student’s t test. Floating bars are min to max, and line is at mean. SPF, specific pathogen free; CD, control diet; mLN, mesenteric lymph node; siLPL, small intestinal lamina propria; TDD, Trp-deficient diet. See also Figure S3.

Considering the profound changes observed in the transcription of genes involved in immune system processes that were microbiota dependent, in particular *Foxp3* and *ll2ra*, we next explored whether dietary Trp deficiency modulated intestinal Tregs. Immune profiling of the SI was performed at 1, 2, and 3 weeks of TDD feeding (Figure 3A). As early as 1 week following dietary Trp depletion, we observed an increase in the frequency of RORγt^+^ Treg cells and a concomitant decrease in Gata3^+^ Treg cells within the small intestinal lamina propria (si-LP), mesenteric lymph nodes (mLN), and colonic lamina propria (cLP) (Figures 3B–3D and S3B). These data together reveal opposing responses in Gata3^+^ Treg cells versus RORγt^+^ Treg cells to the same dietary perturbation, highlighting potentially antagonistic developmental programs analogous to those of effector T helper cell subsets.

Mechanistically, we considered two hypotheses to explain the increased RORγt^+^ Treg cell frequency observed following dietary Trp depletion: (1) microbial signals derived from a TDD might promote the *de novo* differentiation of RORγt^+^ Treg cells, and/or (2) provide mitogenic stimuli that drive the proliferation of pre-existing RORγt^+^ Treg cells. To test these hypotheses, we placed animals on CD or TDD for 3 weeks and assessed the activation and proliferation states of intestinal Treg cell populations. First, substantial increases in the activation state of Tregs isolated from the mLN were observed in TDD-fed mice (Figure 3E). And second, dietary Trp deficiency drove an early burst of proliferation within small intestinal RORγt^+^ Treg cells, which equilibrated over time (Figure 3F). Conversely, the proportion of Ki67^+^ proliferating cells in the Gata3^+^ Treg population was significantly reduced after 3 weeks on a TDD (Figure 3G). In summary, RORγt^+^ Treg cell expansion was driven by an acute proliferative response to signals induced by dietary Trp deficiency to establish a new homeostatic setpoint.

The first consideration to explain our findings was that Tregs are responding to overt inflammation induced by dietary Trp deficiency. To address this, we performed a comprehensive analysis of the intestinal immune compartment of Trp-deficient mice. No significant differences in the abundance of both RORγt^+^Foxp3^−^ Th17 cells and Gata3^+^Foxp3^−^ Th2 cells were detected, except for a slight increase in Th17 cells after 2 weeks of TDD feeding (Figure S3C). On investigation of CD4 T cell activation states in the mLN, mild increases in the frequency of activated CD4^+^FoxP3^−^ conventional T (cT) cells were observed (Figure S3D). We next looked at the capacity for cytokine production from si-LP CD4 T cells, where we observed an expected decrease in IL-17-producing CD4^+^ T cells, but no significant difference in interferon-g-producing CD4^+^ T cells after 3 weeks of TDD feeding. When examining the myeloid compartment, TDD did not alter the proportions of either CXCR3^+^ macrophages or DC populations (Figures S3F and S3G). Induction of RORγt^+^ Treg cells in response to TDD, therefore, was not secondary to overt host inflammation and was indeed the most robust and consistent phenotype observed in response to TDD feeding.

We next examined the effect of TDD feeding on the ILC compartment. This was particularly important, as AhR activity is required to intrinsically promote numbers and function of ILC3 and suppress ILC2 responses.^46,47^ Contrary to these AhR^−/−^ studies, no changes in numbers and function of ILC2 and ILC3 were observed in TDD-fed animals (Figures 3H and 3I).^46–48^ Instead, TDD feeding resulted in greatly reduced proliferative capacity of both ILC2 and ILC3 (Figure 3J). ILC3 require AhR ligands for their function and Tph1 (the rate-limiting enzyme for metabolism of tryptophan into serotonin) regulates ILC2 function and proliferation.^28^ Therefore, the proliferative defects in ILC3 and ILC2 could be explained by loss of AhR and Tph1 activity in the absence of dietary Trp respectively. Nevertheless, these data provide strong evidence that TDD does not precisely phenocopy AhR^−/−^ mice, but is a result of a combination of changes in the microbiota composition and microbial and host metabolites.

### Microbiota are required for Treg cell remodeling induced by dietary Trp deficiency

**Figure 4.**
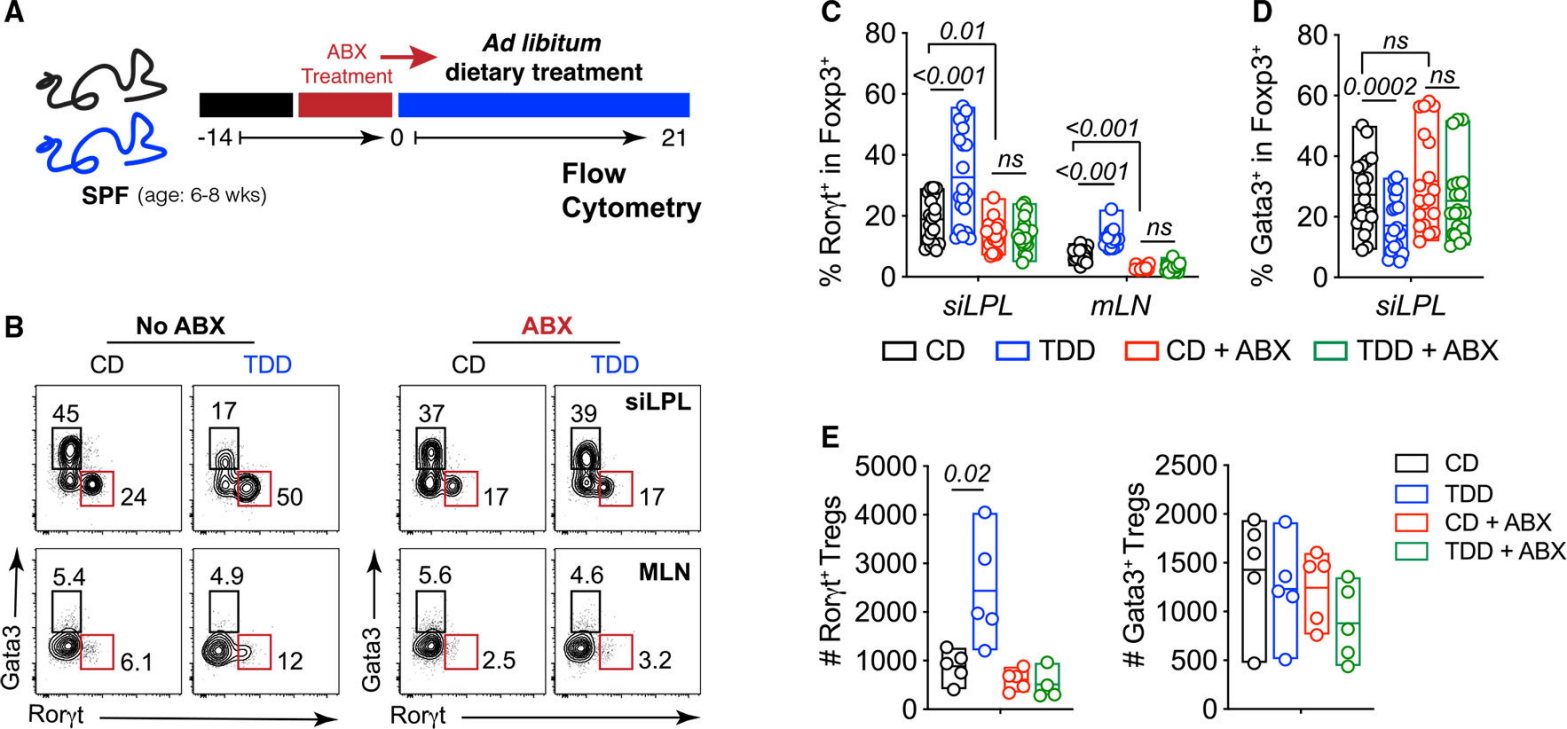
Microbiota mediates the Treg cell changes induced by Trp-deficient diet (A) Schematic for experimental setup. (B) Representative plots and frequencies of Treg populations (gated on TCRb^+^NK1.1^−^CD4^+^Foxp3^+^cells) isolated from SPF (no ABX) or ABX-treated mice fed a CD or TDD as in (A). (C–E) Frequencies (C and D) and numbers (E) of RORγt^+^ Treg cells and Gata3^+^ Treg cells from the indicated organs treated as in (A). Data in (C and D) are pooled from three of four independent experiments (n = 4–5 mice per group). Statistical analysis was performed using two-tailed Student’s t test. Floating bars show min to max, and line is at mean. ABX, pre-treated with antibiotics (ampicillin, gentamycin, vancomycin, metronidazole, and neomycin) to deplete microbiota; CD, control diet; mLN, mesenteric lymph node; siLPL, small intestinal lamina propria; SPF, specific pathogen free; TDD, Trp-deficient diet. See also Figure S4.

Tryptophan is metabolically modified by microorganisms to produce an array of bioactive molecules and several phyla of GI bacteria can induce RORγt^+^ Treg cells.^20,32,49–51^ Based on this literature and our transcriptional profiling data, we hypothesized that metabolites derived from Trp catabolism by bacteria could drive proliferation of RORγt^+^ Treg cells to alter their homeostatic setpoint in TDD-fed mice. CD- and TDD-fed mice were treated with ABX to remove the microbiota (Figures 4A and S4A). Consistent with previous observations,^20,49^ ABX treatment reduced RORγt^+^ Treg cell numbers in CD-fed mice. Notably, the elevated RORγt^+^ and decreased Gata3^+^ Treg cells observed in TDD diet fed mice was ablated by ABX treatment (Figures 4B–4E). Further, no changes in numbers of Th17, ILC3, or ILC2 were observed between control and ABX-treated animals (Figures S4B–S4D). However, the proliferative defect observed in ILC2 and ILC3 isolated from TDD-fed mice remained in ABX-treated mice, suggesting ILC2 and ILC3 proliferation is driven by host-derived Trp metabolism (Figures S4E and S4F). Therefore, we concluded that the Trp-deficient diet modified the microbial community and its metabolites to drive expansion of intestinal RORgt^+^ Treg cells at the expense of Gata3^+^ Treg cells and this was not due to deficiencies in ILCs after TDD feeding.

To further investigate whether specific groups of commensals are sufficient to drive expansion of RORgt^+^ Treg cells in response to TDD feeding, we used individual antibiotics that act on different classes of bacteria. CD- or TDD-fed mice were treated with either metronidazole, which targets anaerobes, vancomycin, which targets mainly gram-positive bacteria, and neomycin, which targets gram-negative bacteria.^52^ Individual administration of neomycin, vancomycin, and metronidazole were all sufficient to block the expansion of RORγt^+^ Treg cells in the absence of dietary Trp (Figure S4G), although metronidazole had an intermediate effect. These data suggest that RORγt^+^ Treg expansion observed was not secondary to general presence of intestinal bacteria, that instead TDD feeding alters a complex interplay between microbial species that cumulatively drive the RORγt^+^ Treg expansion seen in response to TDD feeding.

### Trp-deficient diet results in increased susceptibility to enteric bacterial infection and promotes expansion of RORγt^+^ Treg cells

**Figure 5.**
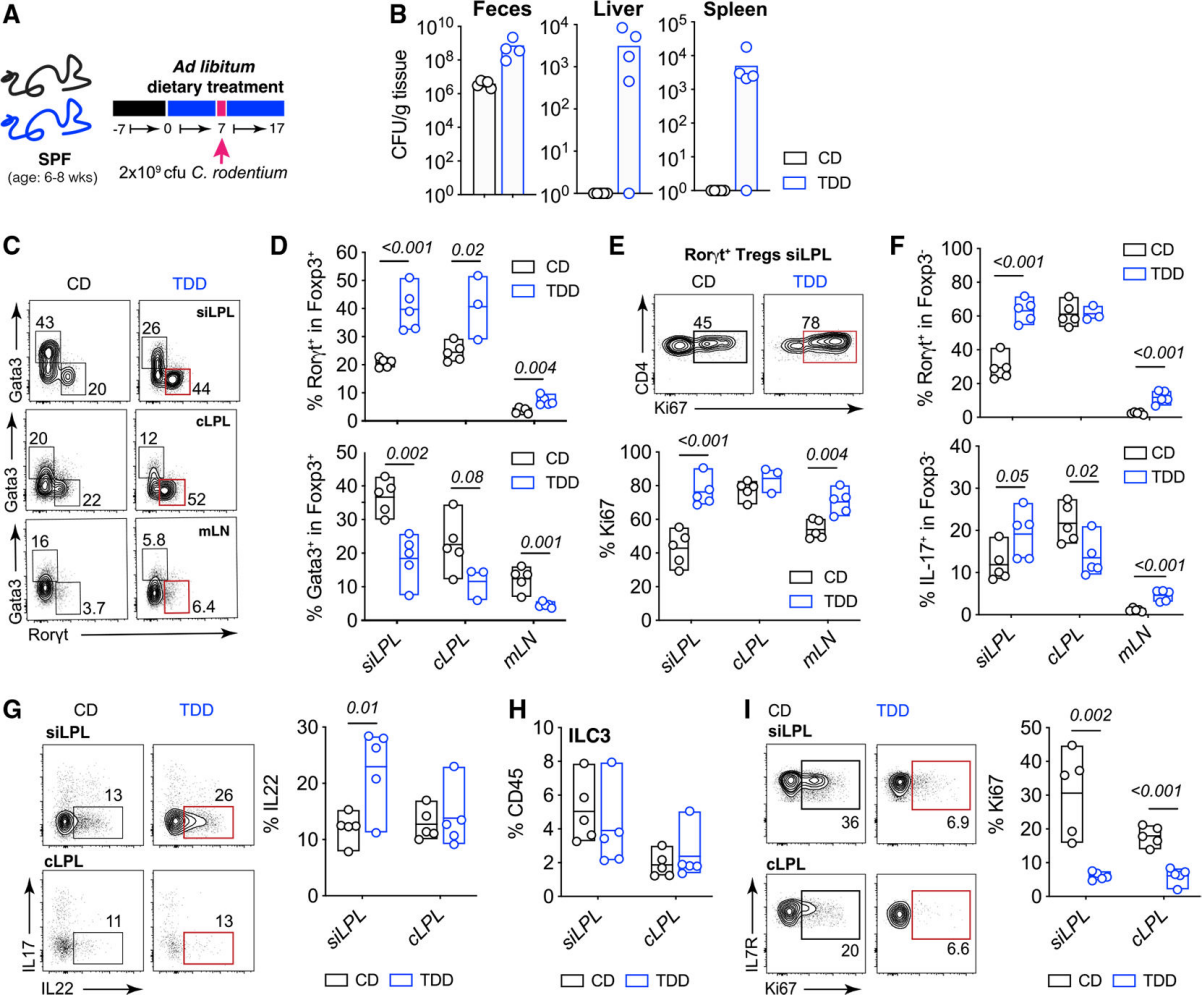
Trp-deficient diet-induced RORγt^+^ Treg cells remain expanded after enteric bacterial infection and result in increased susceptibility to disease (A) SPF mice were fed CD or TDD for 1 week and gavaged with 2 × 10^9^ CFU of *C. rodentium*. (B) Bacterial load in feces, liver, and spleen at day 10 post *C. rodentium* infection on the indicated diets. (C) Data show representative FACs plots and frequencies of Treg populations (gated on TCRb^+^NK1.0031^−^CD4^+^Foxp3^+^cells) isolated from indicated organs and treated as (A). (D) Frequencies of RORγt^+^ (upper) and Gata3^+^ (lower) Treg cells isolated from the siLPL, cLPL, and mLN of mice treated as in (A). (E) Representative FACs plots of CD4 vs Ki67 (upper) and quantification of the frequencies (lower) of Ki67^+^ cells within RORγt^+^FoxP3^+^ Treg cells isolated from the siLPL, cLPL, and mLN at day 10 post *C. rodentium* infection on the indicated diets. (F) Data are frequencies of RORγt^+^ conventional T cells (gated on TCRb^+^NK1.1^–^CD4^+^Foxp3^−^ cells) (upper) and frequencies of interleukin (IL)-17-producing cells (gated on TCRb^+^NK1.1^−^CD4^+^Foxp3^−^) and re-stimulated with PMA/ionomycin together with IL-23 for 3 h (lower). (G) Representative plots and frequencies of IL-22-producing cells within Lin^Neg^, CD127^+^ Rorγt^+^ ILC3 stimulated as in (F). (H) Data show frequency of Lin^Neg^, CD127^+^ Rorγt^+^ ILC3 among CD45^+^ lymphocytes isolated from the si-LP and cLP of CD- or TDD-fed mice at day 10 post *C. rodentium* infection. (I) Representative plots show frequencies of Ki67^+^ cells within ILC3 isolated from the si-LP and cLP of CD- and TDD-fed mice on day 10 post *C. rodentium* infection. Data are representative of two to three independent experiments where n = 4–5 mice per group. Statistical analysis was performed using two-tailed Student’s t test. Floating bars show min to max, and line is at mean. CD, control diet; CFU, colony-forming units; cLPL, colon lamina propria; mLN, mesenteric lymph node; siLPL, small intestinal lamina propria; SPF, specific pathogen free; TDD, Trp-deficient diet.

Several reports have shown that RORγt^+^ Treg cells and Gata3^+^ Treg cells have non-redundant roles in controlling immune responses in the GI tract.^25,49,53,54^ Thus, it was important to establish how the remodeled population of gut Treg cells following TDD feeding responded to intestinal inflammation and whether this phenotype remained during an inflammatory response. We used a bacterial model of intestinal inflammation, *C. rodentium*, to investigate the Treg response to inflammation in TDD-fed animals. This model was chosen because RORγt^+^ Treg cells are reported to suppress the immune response to *C. rodentium*.^55^ Broadly, dietary Trp deficiency resulted in increased bacterial load in the feces and dissemination of bacteria to the liver and spleen at day 10 post-infection (Figures 5A and 5B), showing an inability of TDD-fed mice to control *C. rodentium* infection.

Numerous other factors regulated by Trp metabolites are essential for protection against *C. rodentium*, including ILC3 and Th17 function, and epithelial cell barrier integrity.^27,32,45,56^ Although we found minimal impact on these parameters in naive TDD-fed mice, we next examined the several immune parameters crucial for the control of *C. rodentium* that might be modulated by TDD feeding. Consistent with our earlier data in naive mice, at day 10 post-infection, TDD-fed mice had significantly expanded RORγt^+^ Treg cells with increased proliferation and reduced Gata3^+^ Treg cells (Figures 5C–5E). Surprisingly, the frequency of AhR-dependent Rorγt^+^FoxP3^−^ Th17 cells, essential for protection against *C. rodentium*, was increased in the siLPL and mLN of TDD-fed mice; however, IL-17 production from these cells in the colon, the site of infection, was reduced (Figure 5F). We next examined ILC3 function and numbers after *C. rodentium* infection. Although we did not see any differences in the frequencies of ILC3, nor changes in capacity for IL22 production, there was a drastic reduction in the number of proliferating ILC3 in the si-LP and colon after TDD feeding as observed in naive TDD-fed mice (Figures 5G–5I). This effect was most likely due to loss of AhR ligands available to ILC3 required for their proliferation.^48,57^

Although the effect of dietary Trp deficiency on the intestinal epithelium and the fitness of *C. rodentium* within the lumen itself cannot be ruled out as an explanation for increased susceptibility to infection, these data show that our model of dietary Trp deficiency—that uniquely drives the expansion of Rorγt^+^ T_regs_ and decrease of Gata3^+^ T_regs_—still occurs after inflammation.

### Dietary AhR ligands are sufficient to block Trp deficiency-driven Treg phenotype

**Figure 6.**
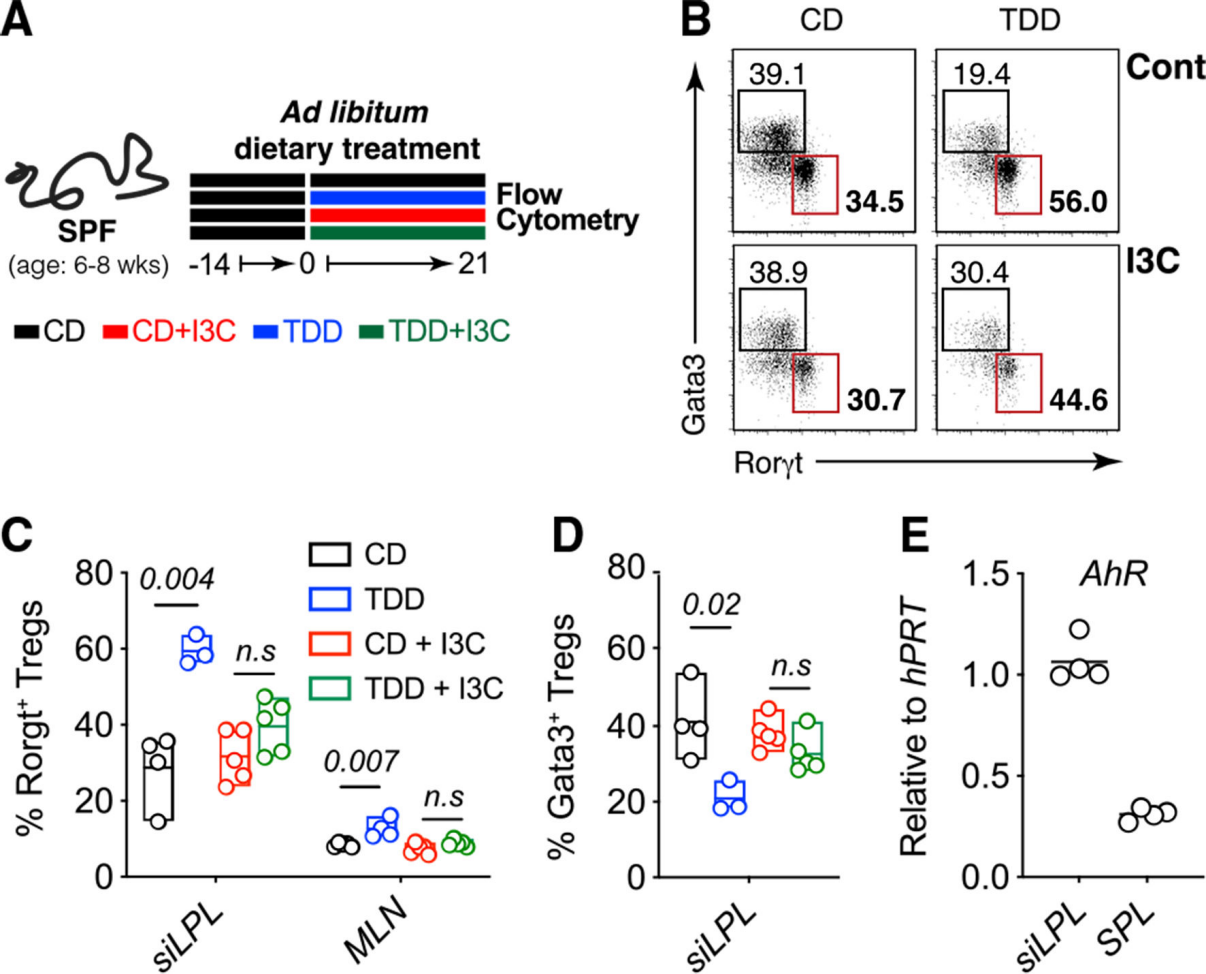
Supplementation with I3C on a Trp-deficient background is sufficient to normalize Treg cell populations (A) SPF mice were fed CD, TDD, CD + 200 ppm I3C or TDD diet + 200 ppm I3Cfor 3 weeks. (B) Representative plots and frequencies of Treg populations (gated onTCRb^+^NK1.1^−^CD4^+^Foxp3^+^cells) in the si-LP isolated from animals treated as indicated. (C and D) Frequencies of RORγt^+^ Treg cells (C) and Gata3^+^ Treg cells (D) within Foxp3^+^ Tregs isolated from the indicated organs of animals fed the indicated diets. Data are representative of two independent experiments (n = 4 mice per group). (E) Relative *AhR* expression (to *hPRT*) within TCRβ^+^CD4^+^Foxp3^–^GFP^+^ Treg cells sorted from the si-LP and spleen of FoxP3-GFP reporter mice. Statistical analysis was performed using two-tailed Student’s t test. Floating bars show min to max, and line is at mean. CD, control diet; I3C, indole-3-carbinol; mLN, mesenteric lymph node; siLPL, small intestinal lamina propria; TDD, Trp-deficient diet. See also Figure S5.

Modification of Trp into AhR ligands is performed by several gut commensals such as *L. reuteri*, which we found were depleted after TDD feeding. We thus hypothesized that metabolism of Trp into AhR ligands by commensal bacteria was influencing levels of RORγt^+^ Tregs and Gata3^+^ Tregs in the GI tract. To test this hypothesis, we asked whether AhR signaling was sufficient to prevent dietary Trp deficiency from remodeling the Treg compartment. The potent AhR ligand, indole-3-carbinol (I3C), was supplemented into our Trp-deficient diet (Figure 6A). I3C provision had no effect on weight loss but was sufficient to restore intestinal RORγt^+^ Treg cells and Gata3^+^ Treg cells to control diet levels on a TDD background (Figures 6A–6D and S5A). To rule out the contribution of other host-derived Trp metabolites, we also investigated the role of serotonin replacement on Treg homeostasis in animals fed TDD (Figures S5B–S5E). We supplied serotonin into the drinking water of CD- and TDD-fed animals, which had no effect on the induction of RORγt^+^ Treg cells (Figures S5B–S5D) or weight loss (Figure S5E) in the absence of dietary Trp. Therefore, we concluded that AhR-reactive metabolites produced by the microbiota regulate the pool of RORγt^+^ and Gata3^+^ Treg cells, either directly or indirectly.

AhR is highly expressed in intestinal Treg populations (Figure 6E) and, indeed, several publications have implicated AhR signaling in regulating intestinal Treg populations.^25,49,58,59^ Treg-specific deletion of AhR results in a reduction of Tregs localized to the intestine,^58^ particularly peripherally induced Nrp^−^ (Rorγt^+^ and Rorγt^−^) Tregs. Interestingly, this phenotype was not observed in complete knockout mice or mice with AhR deleted in all T cells (CD4-Cre). To test whether the loss AhR signaling during TDD feeding was intrinsically required to mediate the expansion of Rorγt^+^ Tregs, *Ahr*^*fl*/*fl*^*Foxp3*^*YFP-Cre*^ and *Ahr*^*fl*/*fl*^CD4-Cre mice were placed on a TDD along with their appropriate controls for 3 weeks. Treg populations were assessed 2 weeks later (Figures S5F and S5G). Although induction of RORγt^+^ Tregs did not reach significance in the *Ahr*^*fl*/*fl*^*Foxp3*^*YFP-Cre*^ cohort, there was a trend toward increased RORγt^+^ Tregs in response to TDD feeding in both AhR-sufficient and -deficient Treg cells (Figure S5F). To further confirm that TDD feeding could induce RORγt^+^ Tregs independent of AhR expression, *Ahr*^*fl*/*fl*^CD4-Cre mice were also placed on a TDD and Tregs assessed. Here, AhR expression within T cells was not required to promote induction of RORγt^+^ Tregs (Figure S5G). Together, these findings indicate that AhR signaling is not acting directly within Tregs, but induction of RORgt^+^ Tregs is mediated through AhR signaling on other gut immune or epithelial cells to suppress Rorγt^+^ Tregs at steady state.

## DISCUSSION

Dietary Trp is an essential amino acid used by both the microbiota and the host to produce critical molecular regulators of homeostasis. Across different phyla, many gut bacterial species make the enzymes required for the synthesis and metabolism of Trp into critical secondary metabolites. For instance, *Lactobacillus reuteri* produces indole-3-lactic acid, which activates AhR to regulate immunological and physiological outcomes.^23,32,34,40^ Indole-3-lactic acid is one of many Trp metabolites whose regulation and function is unknown in the context of dietary Trp deficiency and immune cell metabolism. Notably, *L. reuteri* is strongly decreased in mice with TDD, so it is possible that *L. reuteri* produces one or more Trp metabolites that normally suppress Treg proliferation through AhR signaling. Another possible explanation is that altered microbial diversity in response to a Trp-deficient diet may promote biosynthesis of molecules that activate RORγt^+^ Treg cell proliferation. However, our findings showing reversal of these effects with I3C suggest that the presence of AhR ligands are inhibiting RORγt^+^ Treg cells.

In the context of the high complexity of microbial-derived Trp derivatives and microbial metabolism, our study shows that the metabolic consequence of dietary Trp depletion on the intestinal microbiota leads to a complete alteration of gut Treg homeostasis. This effect is strongest within the SI at the site of nutrient uptake, a unique finding compared with other reports showing the influence of microbial metabolites on colonic Tregs.^19–21^ Most notably, dietary Trp deficiency led to the dysregulation of commensal microbiota within the ileum and the subsequent expansion of RORγt^+^ Treg cells, an effect that depends on the presence of an intact microbiome. Complete reversal of the Treg phenotype induced by dietary Trp depletion was observed through provision of the AhR ligand, indole-3-carbinol, together suggesting that microbially derived AhR ligands inhibit the formation of RORγt^+^ Treg cells.

This report identifies a mechanism by which the microbiota and its metabolites suppress the generation of RORγt^+^ Treg cells. Thus, it is intriguing to speculate that maintenance of RORγt^+^ Treg cells is not only controlled by stimulatory metabolites such as bile acids and SCFAs, but a class of Trp derivatives, produced either by the microbiome or the host, that modulate the dynamic gut Treg compartment. Furthermore, it is evident that during diet-induced Trp deficiency, the expansion of RORγt^+^ Treg cells is at the expense of Gata3^+^ Treg cells. Do RORγt^+^ Treg cells take over the niche of Gata3^+^ Treg cells and thus Gata3^+^ Treg cells lose their fitness over time on a TDD? These findings bring to light the distinct biochemistry regulating the homeostatic control of RORγt^+^ and Gata3^+^ Tregs, which appear to carry out similar functions. Thus, different bacterial metabolic pathways are able influence the composition of the Treg cell population available in the SI to protect against aberrant inflammation. Provision or depletion of immune modifiers in the diet, such as Trp, likely represents a key axis between the microbiome and host to support symbiosis. Understanding the specific Trp derivatives or bacterial species that produce these metabolites that contribute to the balance of RORγt^+^ Treg cells and Gata3^+^ Treg cells in the context of Trp deficiency will be extremely valuable in designing therapies to modulate gut Tregs cells for the treatment of intestinal inflammatory disorders.

### Limitations of the study

Although we have not been able to address the precise cellular mechanism of how AhR activity is regulating intestinal Treg cell subsets in the current study, our data using Treg and T cell-specific AhR conditional knockout animals appear to indicate that AhR ligands are likely acting via a T cell-extrinsic mechanism. While certainly an interesting area for future investigation beyond the scope of the current study, several complex crosses (e.g., *Ahr*^*fl*/*fl*^Villin-Cre or *Ahr*^*fl*/*fl*^CD11c-Cre) could be performed to identify the specific cell type AhR ligands are acting on to drive the observed RORγt^+^ Treg cell expansion phenotype following TDD feeding. Further, while our data clearly demonstrate that animals on a TDD are less able to control *C. rodentium* infection, we note that there are likely several interactions occurring simultaneously between the microbiome, immune system, and intestinal ecosystem that cannot solely be explained by an expansion of RORγt^+^ Treg cells. To this end, dietary Trp deficiency may impact several components of the intestinal microenvironment and microbial niches, potentially altering the ability of *C. rodentium* to survive and thrive in the intestine—in a manner independent of the contribution RORγt^+^ Treg cells have on intestinal immunity. In addition, although our current study has not directly addressed the functional role of intestinal RORγt^+^ Treg cells, we believe that future work using mice wherein RORgt^+^ or Gata3^+^ Treg cells can be specifically depleted will provide further insight into the relative importance of individual intestinal Treg cell subsets. Such model systems could also be used to uncover the different suppressive functions of intestinal Treg cell subsets at steady state and their individual roles following challenge with various intestinal pathogens.

## STAR*METHODS

Detailed methods are provided in the online version of this paper and include the following:

### RESOURCE AVAILABILITY

#### Lead contact

Further information and requests for resources and reagents should be directed to and will be fulfilled by the lead contact, Nicholas Arpaia (na2697@cumc.columbia.edu).

#### Materials availability

All reagents generated or used in this study are available on request from the lead contact with a completed materials transfer agreement. Information on reagents used in this study is available in the key resources table.

### Data and code availability

- RNA sequencing and 16S rRNA sequencing data have been deposited on the publically available (https://www.ncbi.nlm.nih.gov/sites/GDSbrowser/) GEO: GSE222818 and the NCBI BioProject: PRJNA925292 (http://www.ncbi.nlm.nih.gov/bioproject/) databases, respectively. Accession numbers are listed in the key resources table.
- This paper does not report original code. References to all code used are available in the method details section.
- Any additional information required to reanalyze the data reported in this paper is available from the lead contact upon request.

### EXPERIMENTAL MODEL AND SUBJECT DETAILS

#### Mouse strains

Mice were housed and bred in a specific pathogen-free (SPF) barrier facility at Columbia University Irving Medical Center (CUIMC): Wild-type C57BL/6NJ mice were purchased from The Jackson Laboratories, *Ahr*^*fl*/*fl*^ mice^61^ were provided by Richard Blumberg, Harvard Medical School, CD4-Cre, *Foxp3*^*YFP-Cre*^ and *Foxp3*^*GFP*^ mice have been previously described.^62–64^ On arrival to CUIMC, 5–6 week old C57BL/6NJ mice were supplied with dirty bedding from SPF animals bred at CUIMC to normalize microbiota among cages. Cohorts of sex and aged matched 6–10-week-old females (unless otherwise specified) were randomly assigned to experimental groups. Sample sizes were determined from previous experience, existing literature, and statistical power calculations. All mouse experiments were conducted according to USA Public Health Service Policy of Human Care and Use of Laboratory Animals. All protocols are approved by the Animal Care and Use Committee of CUIMC.

#### Diets

Breeding animals were fed breeder chow and F1 offspring were weaned onto standard chow. At 6–8 weeks of age, animals were placed on a control TestDiet Baker’s Amino Acid Diet, 5CC7, with Phylloquinone replacing Menadione as Vitamin K source (see Table 1 for complete diet makeup). After 1–2 weeks, animals were divided into Control or Tryptophan deficient (No Trp) groups. Animals in the No Trp groups were switched to Baker’s Amino Acid Diet with no Tryptophan added. Animals were maintained on these diets for 1–4 weeks. For Indole-3-Carbinol (I3C) supplementation, 200PPM of I3C (sigma) was added to Control and No Trp diets and animals were maintained on this diet for 3 weeks. All diets were manufactured and irradiated by TestDiet.

#### Antibiotic treatment and supplementation of 5-HT

Mice were treated with the following antibiotics in their drinking water: Ampicillin (Sigma-Aldrich) 0.5 g/L; Vancomycin (Gold-Bio) 0.25 g/L; Metronidazole (Sigma-Aldrich) 0.5 g/L; Neomycin 0.5 g/L (Sigma-Aldrich); Gentamycin (Gold-Bio) 0.5 g/L together (AVMNG) or separately at 1 g/L. Drinking water was further supplemented with 2% w/v sucrose for palatability. Water was provided ad libitum for duration of experiment and replaced every 3–4 days to prevent fungal outgrowth and maintain efficacy of the antibiotics. For 5-HT treatment, water was supplemented with 25ug mL^−1^ of 5-HT (Sigma-Aldrich). Water was provided ad libitum for 3 weeks.

### METHOD DETAILS

#### *C. rodentium* infection

8-week-old SPF C57Bl/6 mice were fed CD or TDD diets for 1 week (5 mice per group). The day prior to inoculation, individual colonies of C.rodentium were grown overnight in Luria Bertani (LB). The following day, CFU was measured by OD600 and adjusted to a concentration of 10^10^ CFU/mL. Treated mice were gavaged with 200uL of culture or 2 3 10^9^ CFU of *C.rodentium*. Mice were sacrificed on day 10 of infection. To measure CFU levels in the spleen, liver and feces, tissue was weighed and diluted in appropriate concentration of PBS prior to homogenisation. Serial dilutions of homogenates were then plated on MacConkey agar and incubated overnight. Colonies were then counted to determine CFU/g tissue. Further, colon, terminal ileum and mLN were excised and lymphocytes isolated according to tissue lymphocyte isolation protocol. To elicit cytokine production, lymphocytes were incubated with PMA/Ionomycin (50 ng/mL, 500 ng/mL respectively) and 20ng IL-23 in the presence of Brefaldin A (1 μg/mL) for 3 h. Cells were then stained with the relevant fluorescent antibodies and subject to flow cytometry analysis.

#### 5-HT measurements

Feces were weighed and homogenized in 50 mg/mL enzyme-linked immunosorbent assay (ELISA) standard buffer supplemented with ascorbic acid. Homogenate was then diluted accordingly, and serotonin levels detected by ELISA according to manufacturer’s instructions (Eagle Biosciences).

#### Tissue lymphocyte isolation

For lymphocyte isolation from the GI tract, 12.5 cm of the terminal ileum or the entire colon was excised. Peyers’ patches were removed, and intestinal tissue cut longitudinally, cleaned, and chopped into small pieces. To remove the epithelial layer, intestinal pieces were then placed in “dissociation buffer” containing 5mM EDTA, 2% FCS in PBS and incubated at 37°C for 20 min with shaking. Tissue the incubated with shaking for 45 min in “digestion buffer” containing Collagenase III (Worthington) 1.5 mg/mL, Dispase (Gibco) 0.4 U/mL and DNAase I (Sigma-Aldrich) 0.2 ug/mL in 10% cRPMI followed by 40% Percoll gradient (GE Healthcare). MLN and spleens were removed, and single cell suspensions were prepared by mechanical disruption. For CD4 T cell isolation, the Dynabeads Flowcomp CD4 kit (Fisher Scientific) was used according to the manufacturers protocol.

#### Flow cytometry and cell sorting

Dead cells were excluded by either the fixable Ghost Dye Red 780 (Tonbo Biosciences) or SYTOX Blue Nucleic Acid stain (Invitrogen) for live cell sorting. For flow cytometry, along with anti-CD16/CD32 antibody, the following surface staining monoclonal antibodies were used. Lineage positive cells were excluded to reveal ILC subsets using CD3ε (145-2C11), CD5 (5–7.3), CD19 (6D5), FcεR1 (MAR-1), CD11b (M1/70) and CD11c (N418). For further characterization and delineation of T cell subsets, CD45 (30-F11), NK1.1 (PK136), NKp46 (29A1.4), TCRβ (H57-597), CD4 (RM4–5), IL-7Rα (A7R34) were used. The FoxP3/Transcription Factor staining buffer kit (Tonbo) was used for intracellular staining of FoxP3 (FJK-16s), RORγt (AFKJS), Gata3 (TWAJ), Ki67 (SolA15), IL-17 (Bio17B7) and IL-22 (IL22JOP). All antibodies were purchased from either eBioscience, Biolegend or BD. ILCs were identified as lineage negative (CD3ε, CD5, CD19, FcεR1, CD11b and CD11c)^−^, IL-7Rα^+^ and Gata3^high^ RORγt^−^ (ILC2) or Gata3^Int^ RORgγ^+^ NKp46^+/−^. Tregs were identified as NK1.1^−^TCRβ^+^CD4^+^ FoxP3^+^ and further sub-grouped into Gata3^+^ or RORγt^+^ populations, while Th17 cells were identified as NK1.1^−^TCRb^+^CD4^+^FoxP3−Rorγt^+^ cells.

Flow cytometry was acquired on a custom-configurated LSRFortessa (BD Biosciences) and the FACs Diva software or on a ZE5 Cell analyser (BIO-RAD) and Empress software. All data were analyzed with FlowJo V10.6.1 software (TreeStar). Sort-purified cells were obtained using a FACSAria cell sorter (BD Biosciences).

#### Quantitative PCR analysis of Tregs

CD4 T cells were purified from the spleen using the Dynabeads FlowComp Mouse CD4 Kit and Lamina-propria lymphocytes were isolated from the small intestine of *Foxp3*^*GFP*^ mice. Cells were stained for viability, CD4 and TCRb and sorted on CD4^+^, TCRb^+^, FoxP3-YFP^+^ cells using a FACs Aria. Cells were sorted directly into TRIzol reagent. RNA was purified using phenol-chloroform extraction, and concentration and quality determined using a nanodrop. RNA was first reverse-transcribed to cDNA using qScript cDNA SuperMix (QuantaBio) following the manufacturers protocol. For qPCR, the target gene *Ahr* (F 5′-TCCATCCTGGAAAT TCGAACC-3′; R 5′-TCTTCATCCGTCAGTGGTCTC-3′)was quantified and normalized to the housekeeping gene Hprt (F 5′-CTGGTGAAAAGGACCTCTCGAAG-3′; R 5′-CCAGTTTCACTAATGACACAAACG-3′) using the 2–ΔΔCT method.

#### Quantitative analysis of *L. reuteri*

Ileum intestinal contents or feces were collected and frozen at −80°C. DNA was isolated using phenol-chloroform extraction as previously described in (Farkas et al. 2015). Total DNA concentration was measured using a NanoDrop and normalized across samples. The relative abundance of *L. reuteri* was determined by quantitative PCR using a BioRad CFX384 RealTime System and Syber Green reagent. Cycling conditions were 95C for 10 min, 95°C for 15 s and 60°C for 60 s for 40 cycles: melting curve 60°C–95°C. The following *L. reuteri* specific primers were used – 5′-ACCGAGAACACCGCGTTATTT-3′ (Fwd); 5′-CATAACTTAACCTAAACAAT CAAA-3′ (Rev). L. reuteri CT values were normalized to total bacteria (Eubacteria) using 16S rDNA primers 5′-ACTCCTACGGGAGG CAGCAG-3′ (Fwd); 5′-ATTACCGCGGCTGG-3′ (Rev) using the 2–ΔΔCT method.

#### 16S rRNA microbiome analysis

Small intestinal contents were collected from the terminal ileum by gently squeezing out the contents with a sterile tip. Feces and ileal contents were frozen at −80 for storage. DNA was extracted using the Mo-Bio MagAttract PowerSoil kit. The V3-V4 regions of the 16S rDNA gene were PCR amplified using individually barcoded primers on the MiSeq V3 2×300 platform.

For generating amplicon sequence variant table (ASV) table ASVs, Divisive amplicon denoising algorithm version 2 (DADA2 1.12.1)^66^ was used for quality-filtered, trimmed, error correction, exact sequence inference, chimera removal. Taxonomic classification was performed using a native naïve RDP Bayesian classifier against the Greengenes database (97% cutoff). The table was imported into R 3.6.1 to analyze for β-diversity (unifrac) and were performed using a function of the phyloseq v1.28.0 package.^67^ Differential abundance analysis for bacterial ASVs was performed using DESeq2.^68^ The p values were adjusted using the Benjamini-Hochberg method for controlling the false discovery rate.

#### RNA sequencing analysis

For RNA sequencing analysis 7-week-old female SPF or ABX-treated C57BL/6 mice were fed CD or TDD for 3 weeks. At endpoint, a 0.5 cm of small intestinal ileum tissue was cut 2cm from the base of the cecum. Tissue was opened longitudinally, briefly washed in ice-cold PBS prior to snap freezing in liquid nitrogen. Frozen tissue was homogenized, and RNA extracted using the Qiagen RNEasy miniprep kit as per the manufacturer protocol. RNA concentration was measured using a bioanalyzer. RNA-sequencing was performed at the JP Sulzberger Columbia Genome Center. Poly-A^+^ mRNAs were enriched from total RNA using oligo d(T) beads, followed by cDNA library construction. Libraries were then sequenced using an Illumina NovaSeq 6000. Sequencing delivered 40 million reads per sample on average. Reads were aligned to the *Mus musculus* reference transcriptome (Mouse:GRCm38) using kallisto (0.44.0). Differential expression analysis was performed using the integrated web application iDEP.91.^65^ Genes expression was called with 0.5 counts per million. Differentially expressed genes were called using an FDR cutoff of 0.05 with a minimal fold change of 2. Pathway analysis was carried out using STRING and metascape online analysis software.

#### CE-TOFMS analysis

Fecal samples were snap frozen and sent to Human Metabolome Technologies (HMT). At HMT, samples were mixed with 50% acetonitrile in water (v/v) containing internal standards (10 μM) and homogenized and filtrated through 5-kDa cut-off filter (ULTRAFREE-MC-PLHCC, Human Metabolome Technologies, Yamagata, Japan) to remove macromolecules. The filtrate was centrifugally concentrated and resuspended in 50 μL of ultrapure water immediately before the measurement. The compounds were measured in the Cation and Anion modes of CE-TOFMS based metabolome analysis.

Peaks detected in CE-TOFMS analysis were extracted using automatic integration software (MasterHands ver. 2.17.1.11 developed at Keio University) to obtain peak information including m/z, migration time (MT), and peak area. The peak area was then converted to relative peak area and the peak detection limit was determined based on signal-noise ratio; S/N = 3. Metabolites were then assigned from HMT’s standard library and Known-Unknown peak library based on m/z and MT. The tolerance was ±0.5 min in MT and ±10 ppm in m/z. If several peaks were assigned the same candidate, the candidate was given the branch number. Hierarchical cluster analysis (HCA) and principal component analysis (PCA) were performed by statistical analysis software (developed at HMT). The profile of peaks with putative metabolites were represented on metabolic pathway maps using VANTED (Visualization and Analysis of Networks containing Experimental Data) software. Hierarchical cluster analysis (HCA) and principal component analysis (PCA) were performed by statistical analysis software (developed at Human Metabolon Technologies).

### QUANTIFICATION AND STATISTICAL ANALYSIS

p value datasets were determined by paired or unpaired Student’s t-test with 95% confidence interval. Normal distribution was assumed. For metabolomic analysis where equal variances could not be assumed, Welch’s correction was performed. Statistical tests were performed with GraphPad V8 software (GraphPad software inc).

## Supplementary Material

1

## Figures and Tables

**Table 1.** Control and tryptophan-deficient diet ingredients

Ingredient	Control (%)	No Trp (%)

Corn starch	41.7824	41.7824

Sucrose	25.9000	25.9000
Baker Amino Acid Premix	16.00	16.00

Arginine	0.83	0.83
Histidine	0.49	0.49
Isoleucine	0.80	0.80
Leucine	1.20	1.20
Lysine	1.10	1.10
Methionine	0.60	0.60
Cystine	0.40	0.40
Phenylalanine	0.80	0.80
Tyrosine	0.40	0.40
Threonine	0.78	0.78
Tryptophan	0.20	0.000
Valine	0.80	0.80
Alanine	1.00	1.00
Aspartic Acid	1.00	1.00
Glutamic Acid	1.00	1.00
Glycine	0.99	0.99
Proline	1.00	1.00
Serine	1.00	1.00
Taurine	0.00	0.00

Baker Amino Acid Mineral Premix	10.000	10.000

Corn oil	5.000	5.000

Sodium bicarbonate	1.000	1.000

Baker Amino Acid Vitamin Premix w/Phylloquinone	0.2000	0.2000

Choline chloride	0.1000	0.1000

Ethoxyquin (a preservative)	0.0136	0.0136

DL-alpha Tocopheryl acetate (vitamin E)	0.0046	0.0046

**KEY RESOURCES TABLE:** 

REAGENT or RESOURCE	SOURCE	IDENTIFIER

Antibodies		

Purified anti-mouse CD16/CD32	Tonbo	Cat#70–0161; RRID: AB_2621487
BV786 Rat Anti-Mouse CD45	BD Biosciences	Cat#564225; RRID: AB_2716861
anti-mouse Fc epsilon Receptor I alpha biotin	BioLegend	Cat#134303; RRID: AB_1626100
anti-mouse CD5 biotin	BioLegend	Cat#100603; RRID: AB_312732
anti-mouse CD19 biotin	BioLegend	Cat#115503; RRID: AB_313638
anti-mouse CD3e biotin	BioLegend	Cat#100303; RRID: AB_312668
anti-mouse NK1.1 BUV395	BD Biosciences	Cat#564144; RRID: AB_2738618
anti-mouse TCRb BV711	BD Biosciences	Cat#563135; RRID: AB_2738023
anti-mouse CD11b BV510	BD Biosciences	Cat#562950; RRID: AB_2737913
anti-mouse CD11c BV510	BD Biosciences	Cat#562949; RRID: AB_2732056
anti-mouse CD4 BUV737	BD Biosciences	Cat#612844; RRID: AB_2870166
anti-human/mouse RORgt PE	eBioscience	Cat#12-6988-82; RRID: AB_1834470
anti-mouse/rat Foxp3 FITC	eBioscience	Cat#71-5775-40; RRID: AB_469975
anti-mouse/rat Foxp3 eFluor450	eBioscience	Cat#48-5773-82; RRID: AB_1518812
anti-mouse Gata-3 eFlour660	eBioscience	Cat# 50-9966-42; RRID: AB_10596663
anti-mouse CD127 PE-Cy7	Tonbo	Cat#60-1271;; RRID: AB_2621859
anti-mouse/rat Ki-67 eFluor450	eBioscience	Cat#48-5698-82; RRID: AB_11149124
anti-mouse IL-22 APC	eBioscience	Cat#16-7222-82; RRID: AB_2016695
anti-mouse/rat IL-17A eFluor450	eBioscience	Cat#48-7177-82; RRID: AB_11149503
anti-mouse IFNg BV786	BD Biosciences	Cat#563773; RRID: AB_2645029
anti-mouse TCRb PerCP-Cy5.5	Tonbo	Cat # 65-5961; RRID: AB_2621911
anti-mouse CD45 (pan) BUV395	BD Biosciences	Cat#564279 RRID: AB_2651134
anti-mouse CD11c violetFluor450	Tonbo	Cat#75-0114; RRID: AB_2621937
anti-mouse Ly-6C BV510	BioLegend	Cat#128033; RRID: AB_2562351
anti-mouse/human CD11b BV650	BioLegend	Cat#101239; RRID: AB_1112557
anti-mouse CD103 FITC	BioLegend	Cat#121419; RRID: AB_10709438
anti-mouse MHC Class II (I-A/I-E) PerCP-Cy5.5	BioLegend	Cat#107625; RRID: AB_2191072
anti-mouse CD64 (FcgRI) APC	BioLegend	Cat#139305; RRID: AB_11219205
anti-mouse IgA biotin	BioLegend	Cat#407003; RRID: AB_315078
anti-mouse NK1.1 biotin	BioLegend	Cat#108703; RRID: AB_313390
anti-mouse CD62L (L-Selectin) PercP-Cy5.5	BioLegend	Cat#100431; RRID: AB_2187123
anti-human/mouse CD44 redFluor710	Tonbo	Cat#80-0441; RRID: AB_2621985
Ghost Dye Red 780	Tonbo	Cat#13-0865
Streptavidin BUV496	BD Biosciences	Cat#612961; RRID: AB_2870237
Streptavidin PE-Cy7	BioLegend	Cat#108703
Sytox Blue viability dye	ThermoFisher	Cat#S34857

Bacterial and virus strains		

Citrobacter rodentium	Ivaylo Ivanov	Ladinsky et al., Science^60^

Chemicals, peptides, and recombinant proteins		

Collagenase Type III	Worthington Biochemical	Cat#NC9405360
Dispase II Powder	Gibco	Cat#17-105-041
DNAse 1, Grade II	Sigma-Aldrich	Cat#10104159001
HEPES	Fisher Scientific	Cat#15-630-080
Percoll	GE Healthcare	Cat#GE17-0891001
Indole-3-Carbinol	Sigma-Aldrich	Cat#17256
TRIzol Reagent	Fisher Scientific	Cat#15-596-018
lonomycin Calcium Salt (from streptomyces)	Sirma-Aldrich	Cat#I0634
Phorbol 12-Myristate 13-Acetate	Sigma-Aldrich	Cat#P8139
Brefaldin A, natural	Thermo Scientific	Cat#AC297140050
Serotonin hydrochloride (5-HT), powder	Sigma-Aldrich	Cat#H9523
Ampicillin Sodium Salt	Sigma-Aldrich	Cat#A9518
Neomycin Sulfate	Sigma-Aldrich	Cat#4801
Vancomycin Hydrochloride	Gold Biotechnology	Cat#V200
Metronidazole	Sigma-Aldrich	Cat#M1547
Gentamicin Sulfate	Gold Biotechnology	Cat#400-25
Sucrose	Sigma-Aldrich	Cat#S0389

Critical commercial assays		

Foxp3/Transcription Factor Staining Buffer Kit	Tonbo	Cat#TNB-0607-KIT
Mo-Bio MagAttract PowerSoil kit	Qiagen	Cat#2700-4-KF
Qiagen RNEasy miniprep kit	Qiagen	Cat#74104
Dynabeads^TM^ FlowComp^TM^ Mouse CD4 Kit	Invitrogen	Cat#11461D
BD Cytofix/Cytoperm Fixation/	BD Biosciences	Cat#554714
Permeabilization Solution Kit		
Eagle Biosciences Inc Serotonin	Fisher Scientific	Cat#50-814-05
Ultrasensitive ELISA		
qScript cDNA SuperMix	QuantaBio	Cat#101414-108
SYBR Green qPCR Master Mix (2X)	Thermo Scientific	Cat#K0253

Deposited data		

RNA Sequencing	This Paper	GEO: GSE222818
16s rRNA sequencing	This Paper	BioProject: PRJNA925292; http://www.ncbi.nlm.nih.gov/bioproject/925292

Experimental models: Organisms/strains		

C57BL/6NJ	The Jackson Laboratory	Cat#005304
Ahr^fl/fl^	Richard Blumberg	Walisser et al., PNAS 2005^61^
FoxP3^YFP^–^Cre^	Alexander Rudensky	Rubtsov et al., Immunity 2008^62^
CD4-Cre	Alexander Rudensky	Sadawa et al., Cell 1994^63^
FoxP3-GFP	Alexander Rudensky	Fontenot et al., Nat Immunol^64^

Oligonucleotides		

*L. reu* F 5’-ACCGAGAACACCGCGTTATTT-3’	N/A	Zelante et al.^32^
*L. reu* R 5’-	N/A	Zelante et al.^32^
CATAACTTAACCTAAACAATCAAAGATTGTCT-3’		
*EuBac* F 5’-ACTCCTACGGGAGGCAGCAG-3’	N/A	Zelante et al.^32^
*EuBac* R 5’- ATTACCGCGGCTGCTGG-3’	N/A	Zelante et al.^32^
*AhR* F 5’-TCCATCCTGGAAATTCGAACC-3’	N/A	Zelante et al.^32^
*AhR* R 5’-TCTTCATCCGTCAGTGGTCTC-3’	N/A	Zelante et al.^32^
*Hprt* F 5’ -CTGGTGAAAAGGACCTCTCGAAG-3’	N/A	This Paper
*Hprt* R 5’- CCAGTTTCACTAATGACACAAACG-3’	N/A	This Paper

Software and algorithms		

Flowjo version 10	N/A	https://www.flowjo.com
GraphPad Prism	N/A	https://www.graphpad.com/scientific-software/prism/
Adobe Illustrator	N/A	https://www.adobe.com/
iDEP.91	N/A	http://bioinformatics.sdstate.edu/idep90/ ^65^
Metascape − A Gene Annotation + Analysis Resource	N/A	https://metascape.org/gp/index.html#/main/step
String Protein-Protein Interaction Networks	N/A	https://string-db.org
DADA2 1.12.1	N/A	Callahan et al., Nature Methods 2016^66^
phyloseq v1.28.0 package	N/A	Quest et al., Nuc Acids Res 2013^67^
DESeq2	N/A	McMurdie et al., PLoS ONE 2013^68^
MasterHands ver. 2.17.1.11	N/A	N/A

Other		

BD LSR Fortessa Flow Cytometer	BD	N/A
Aria II	BD	N/A
Bio-Rad ZE5	BioRad	N/A
MiSeq V3 2x300 platform	Illumina	N/A
Illumina NovaSeq 6000	Illumina	N/A
Zirconia/Silica Beads 0.1mm	Fisher Scientific	N/A
Test Diet Amino Acid Diet with Phylloquinone as Vitamin K Irradiated	W.F. Fisher & Son (Test Diet)	Cat#9GW4
Test Diet Baker’s Amino Acid Diet w/no added TRP and Phylloquinone as Vitamin K Source Irradiated	W.F. Fisher & Son (Test Diet)	Cat#9GW5
Test Diet Amino Acid Diet with Phylloquinone as Vitamin Kand 0.02% Indole-3-Carbinole Irradiated	W.F. Fisher & Son (Test Diet)	Cat#5WK2
Test Diet Baker’s Amino Acid Diet w/no added TRP and Phylloquinone as Vitamin K Source Irradiated	W.F. Fisher & Son (Test Diet)	Cat#5WK3

## Highlights

- Trp deficiency alters the balance of intestinal Treg subsets, expanding RORγt^+^ Treg
- Trp-deficient diet drives microbiota-dependent transcriptional changes in the ileum
- Trp-deficient diet expansion of RORγt^+^ Treg occurs in a microbiota-dependent manner
- AhR ligand provision restores RORγt^+^ Treg balance in the absence of dietary Trp
