# Supplementary material for: Dietary tryptophan deficiency promotes gut RORγt+ Treg cells at the expense of Gata3+ Treg cells and alters commensal microbiota metabolism
Source: Cell Rep. Author manuscript; Available in PMC 2023 May 1. (PMC10150404; doi:10.1016/j.celrep.2023.112135)
Supplement: 1 [file NIHMS1887260-supplement-1.pdf]

Cell Reports, Volume *42*

Supplemental information

Dietary tryptophan deficiency promotes gut RORγt⁺ Treg cells at the expense of Gata3⁺ Treg cells and alters commensal microbiota metabolism

Lucille C. Rankin, Katherine A. Kaiser, Kenia de los Santos-Alexis, Heekuk Park, Anne-Catrin Uhlemann, Daniel H.D. Gray, and Nicholas Arpaia

Rankin *et. al.* Figure S1

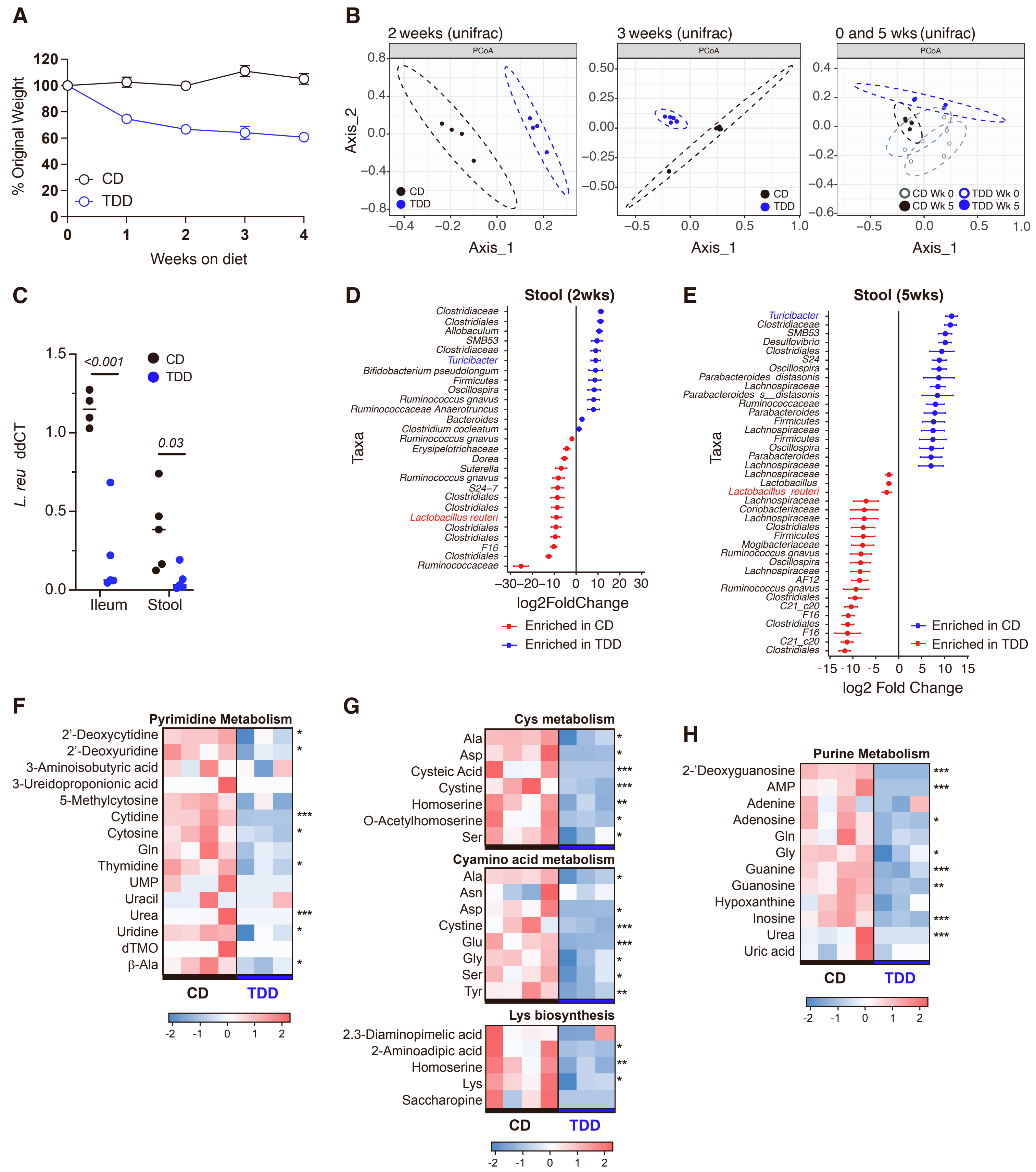

Supplementary Figure 1. Faecal microbiota and metabolite changes after Trp deficient diet feeding, related to Figure 1.

16s rRNA sequencing was performed on the stool of mice fed CD (black) or TDD (blue) for 0, 2, 3 and 5 weeks. (**a**) shows weights over time of SPF mice fed CD or TDD for four weeks. (**b**) is a weighted unifrac PCoA plot representing the differences in the beta diversity of bacterial communities at the indicated weeks on diets. (**c**) shows relative abundance of *L. reuteri* in the ileum and stool of mice placed on a CD and TDD for 3 weeks determined by qPCR analysis. Data is representative of 2 experiments (*n*=4-5 mice per group/experiment). (**d and e**) are forest plots showing Log_2FC of the most differentially abundant taxa between animals fed CD or TDD within the stool at 2 (**d**) and 5 (**e**) weeks on the indicated diets. Differential metabolome analysis was performed on the stool of CD and TDD fed mice using CE-TOFMS (**f-h**). The relative abundance of metabolites in the stool of CD or TDD fed mice that are involved in amino acid (**f**) purine (**g**) and pyrimidine (**h**) metabolism are represented by heatmaps. The p-value is computed by Welch's t-test. (*<0.05, **<0.01, ***<0.001). Heatmaps were generated using standardized value of relative area in detected peaks, shown in legend. Data is from one independent experiment (*n*=3-4 mice/group). CD, control diet; TDD, Trp deficient diet; CE-TOFMS, Capillary Electrophoresis Time-of-Flight Mass Spectrometry.

Rankin *et. al.* Figure S2

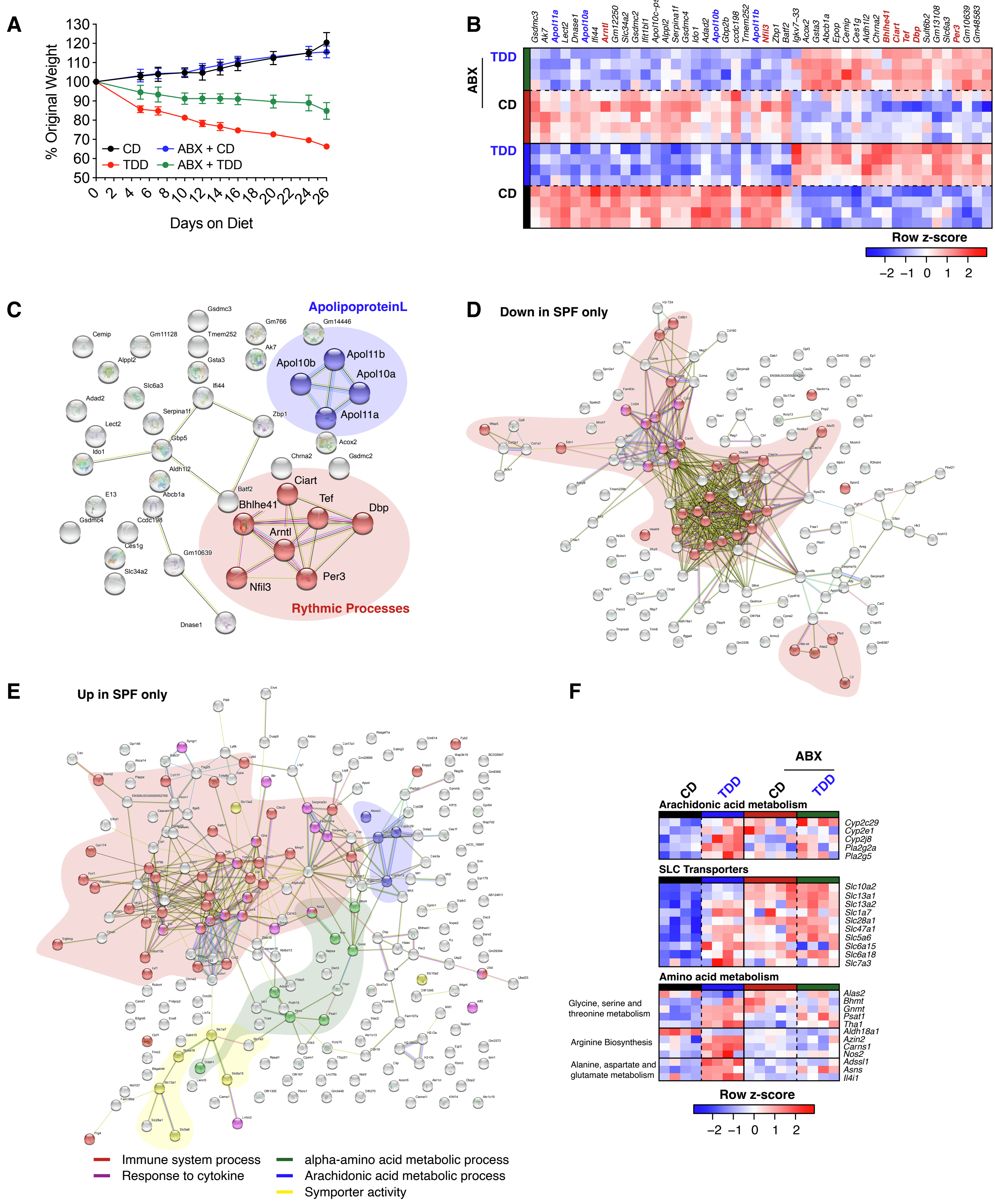

Supplementary Figure 2. Transcriptional changes within the ileum after dietary Trp deficiency, related to Figure 2.

SPF mice were fed CD and placed on ABX to deplete microbiota before transferring to TDD for 3 weeks. Intestinal ileum from mice treated as in (**Figure 2a**) was subject to RNA sequencing analysis. (**a**) is weight loss of SPF mice fed CD, SPF mice fed TDD, ABX-treated mice fed CD and ABX-treated mice fed TDD. A heatmap for genes that were differentially regulated in both SPF and ABX treated animals after TDD feeding is shown in (**b**). (**c-e**) are string protein network analysis of protein-protein interactions using (**c**) genes differentially regulated in both SPF and ABX treated mice, (**d**) downregulated in TDD diet treated SPF mice and (**e**) upregulated in TDD diet treated SPF mice. (**f**) shows a heatmap of selected metabolic and SLC transporter gene expression profile within the ileum of mice treated as indicated. SPF, specific pathogen free; CD, control diet; TDD, Trp deficient diet; ABX, pre-treated with antibiotics (ampicillin, gentamycin, vancomycin, metronidazole, and neomycin) to deplete microbiota.

Rankin *et. al.* Figure S3

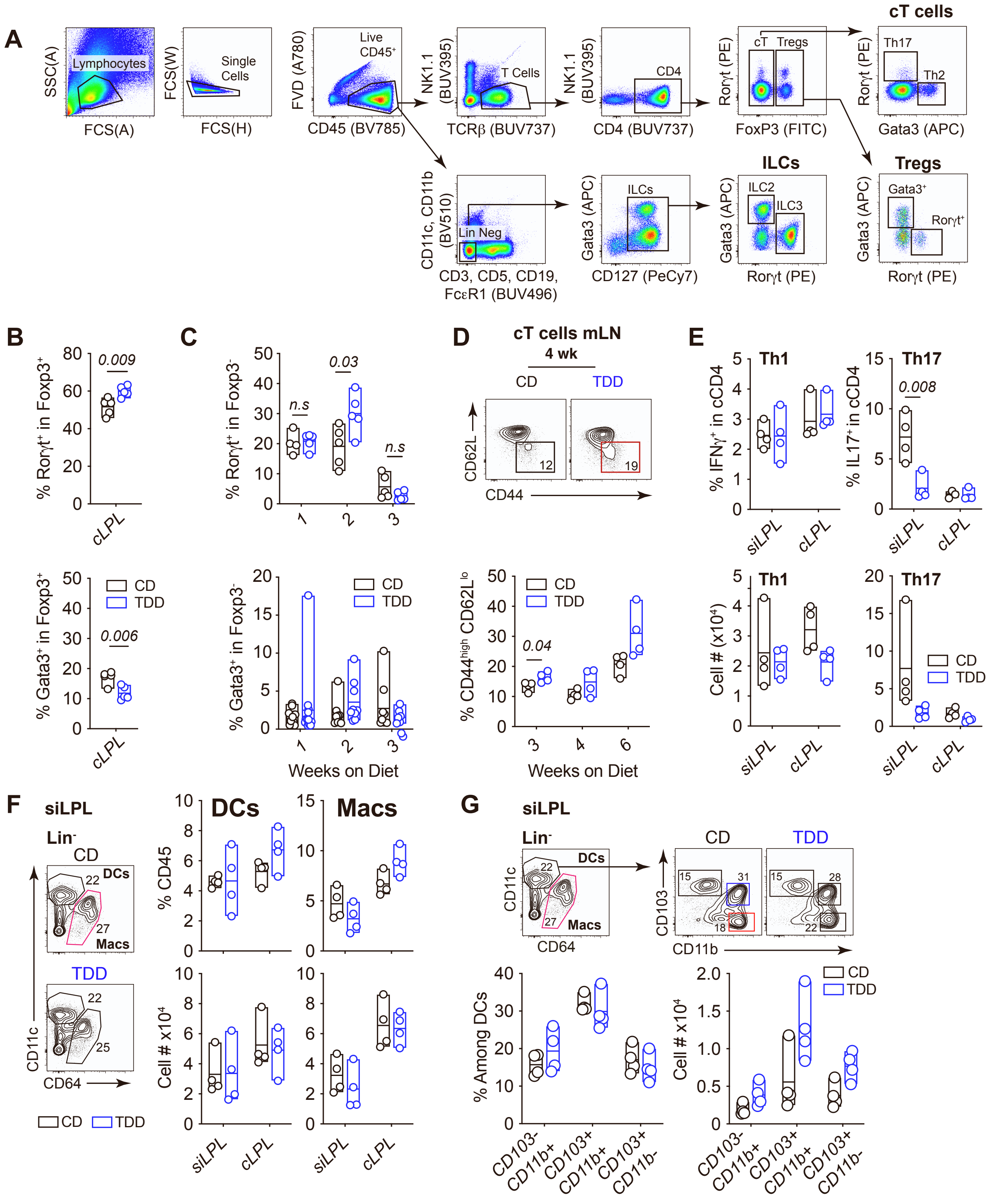

Supplementary Figure 3. The impact of dietary Trp deficiency on the lymphocyte compartment of the GI tract , related to Figure 3.

(**a**) are representative flow cytometry plots of lymphocytes isolated from the si-LPL to show the gating strategy used to delineate lymphocyte populations (**b-e**). SPF mice were fed CD for 2 weeks and switched to TDD or maintained on CD for a further 1-6 weeks. Frequencies are shown of $ROR\gamma t^{+}$ and $Gata3^{+}$ (**b**) Treg cells isolated from the cLPL of mice fed CD (black) or TDD (blue) for 1 week. (**c**) shows frequency of $ROR\gamma t^{+}$ Th17 cells (upper) and $Gata3^{+}$ Th2 cells (lower) within $CD4^{+}$ $FoxP3^{-}$ cT cells from the si-LP at the indicated weeks on CD or TDD. Data is representative of at least two independent experiments (*n*=3-5 mice/group). Data shown in (**d**) shows representative FACS plots (upper) of CD44 vs. CD62L expression in $CD4^{+}FoxP3^{-}$ conventional T cells (cT) isolated from the mLN. Graphs show frequencies of activated cT cells at the indicated weeks on CD or TDD (lower). Data in (**e**) shows frequencies (upper) and numbers (lower) of IFNγ (left) IL17 (right) producing CD4 T cells. (**f**) and (**g**) show numbers and frequencies of myeloid cell populations isolated from the si-LP of mice fed control or TDD for 3 weeks. Representative FACs plots in (**f**) shows expression of CD11c and CD64 amongst Lin Neg cells (TCRb, IgA, NK1.1 Neg). Graphs show enumeration of frequencies (upper) and numbers (lower) of DC ($CD11c^{hi}CD62L^{lo}$) and macrophages ($CD11c^{int}CD62L^{hi}$) under indicated conditions. (**g**) upper panel shows FACs plots of DC populations gated as (**f**) and delineated by CD103 and CD11b expression. Lower panels are frequencies (left) and numbers (right) of indicated DC populations. Data is representative of 2 independent experiments (*n*=4-5 mice/experiment). Statistical analysis was performed using two-tailed student's t-test. Floating bars are min to max, and line is at mean. SPF, specific pathogen free; CD, control diet; TDD, Trp deficient diet; siLPL; small intestinal lamina propria; cLPL, colon lamina propria; mLN, mesenteric lymph node; ABX, pre-treated with antibiotics (ampicillin, gentamycin, vancomycin, metronidazole, and neomycin) to deplete microbiota.

Rankin *et. al.* Figure S4

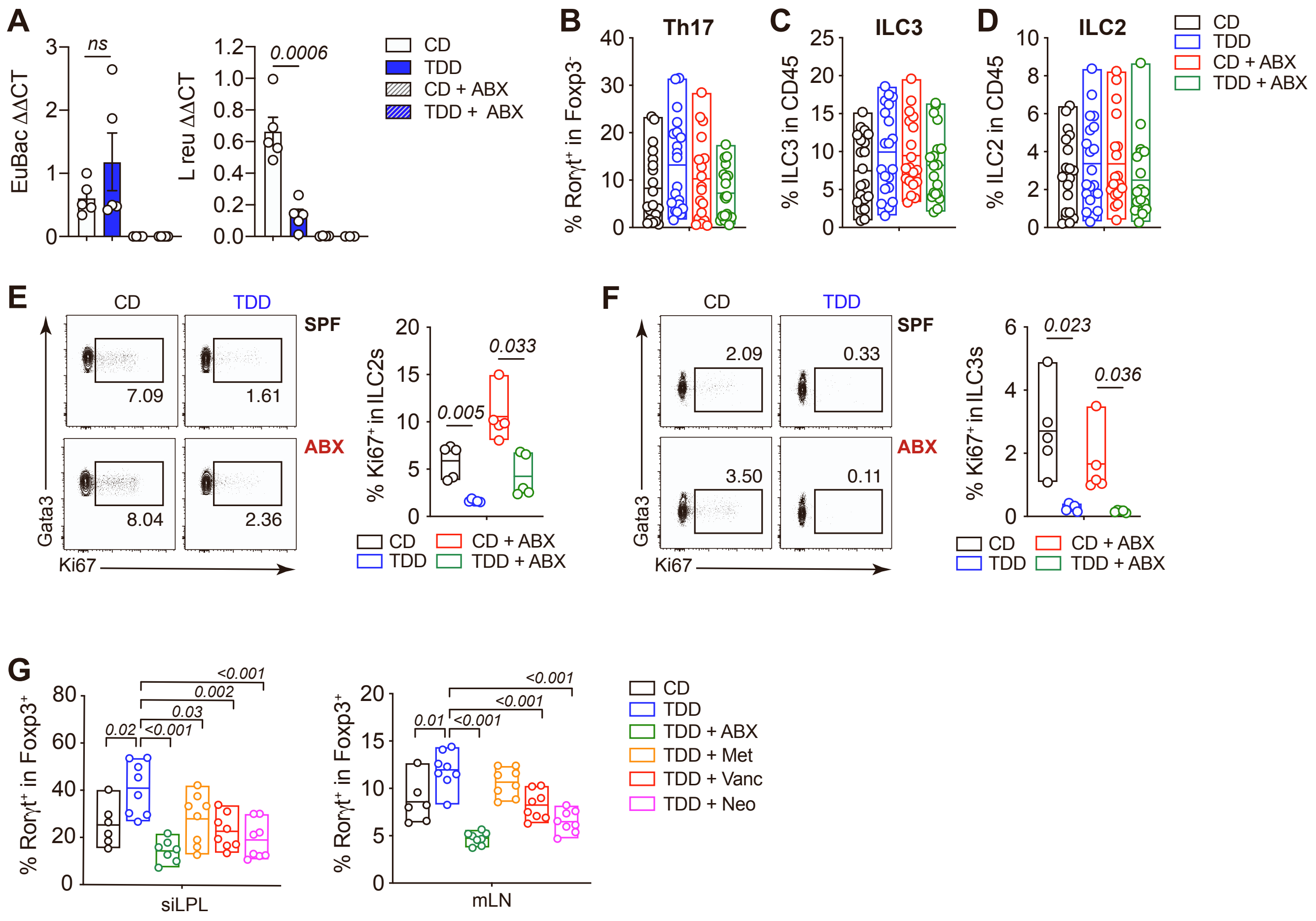

Supplementary Figure 4. Microbiota is required for acute Trp-deficiency to promote expansion of intestinal RORγt^{+} Treg cells and loss of Gata3^{+} Treg cells, related to Figure 2.

SPF mice fed CD, SPF mice fed TDD, ABX-treated mice fed TDD, and ABX-treated mice fed TDD as in (**Figure 4a**) were analysed for Treg populations in the siLPL. (**a**) is a 16S qPCR analysis of DNA from the stool of mice treated as specified using probes against pan-bacterial (left) and *L.reu* specific (right) probes. (**b-d**) are frequencies of CD4^{+}Foxp3^{-}RORγt^{+} Th17 cells. (**b**) ILC3 (**c**) and ILC2 (**d**). Data in (**b-d**) is pooled from three of four independent experiments (*n* = 4-5 mice per group). (**e and f**) show representative plots and enumeration of Ki67 expression within ILC2 (**e**) and ILC3 (**f**) isolated from the siLPL under the indicated conditions. Data is representative of 2 independent experiments (*n* = 4-5 mice per group). (**g**) CD fed mice were treated with an ABX cocktail (ABX) or individual antibiotics metronidazole (met), vancomycin (Vanc), and neomycin (Neo) prior to bring placed on a TDD for 3 weeks. Data in (**g**) show frequencies of RORγt^{+} Treg cells isolated from the siLPL (left) and mLN (right) of mice treated as indicated for three weeks. Data in (**f**) is pooled from two independent experiments (*n* = 3-5 mice per group). Statistical analysis was performed using two-tailed student's t-test. Floating bars show min to max, and line is at mean. SPF, specific pathogen free; CD, control diet; TDD, Trp deficient diet; siLPL, small intestinal lamina propria; mLN, mesenteric lymph node.

Rankin *et. al.* Figure S5

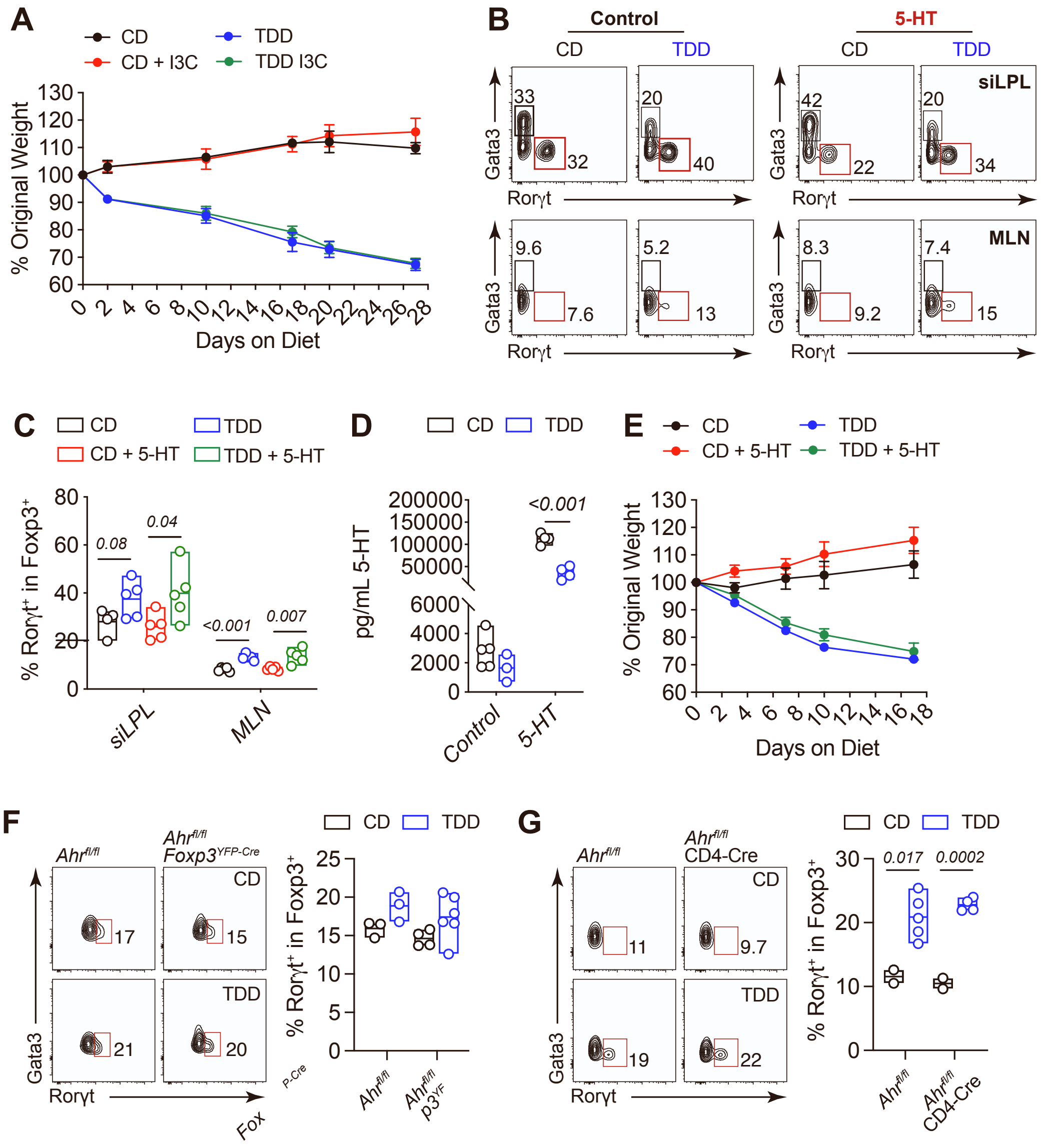

Supplementary Figure 5. Dietary Trp deficiency mediated induction of RORγt$^+$ Treg cells and weight loss is independent of oral supplementation with 5-HT and is not Treg intrinsic, related to Figure 6.

(**a**) is a graph of weights over time of SPF mice fed CD, TDD, CD + 200ppm I3C or TDD diet + 200ppm I3C for four weeks. (**b,c**) SPF mice were fed CD or TDD and treated with 25ug 5-HT in the drinking water for 3 weeks. (**b**) are representative plots of Treg populations (gated on TCRb$^+$NK1.1$^-$ CD4$^+$Foxp3$^+$cells) in the siLPL and mLN isolated from animals treated as indicated and enumerated in (**c**). Faecal 5-HT levels are shown in (**d**) from SPF CD and TDD fed mice treated with or without or 25ug 5-HT in the drinking water for three weeks. (**e**) shows weights over time of SPF control or 5-HT treated mice fed CD or TDD for three weeks. Data representative of two independent experiments (*n* = 4-5 mice per group). Groups of SPF *AhR^{flfl}* and *Ahr$^{fl/fl}$Foxp3$^{YFP-Cre}$* (**f**) or *AhR^{flfl}* and *Ahr$^{fl/fl}$*CD4-Cre (**g**) mice were fed CD or TDD for 3 weeks. Representative plots show RORγt$^+$ cells within FoxP3$^+$ Tregs isolated from the mLN of mice fed CD or TDD. Graphs in (**f**) and (**g**) show frequencies of RORγt$^+$ cells within mLN FoxP3$^+$ Tregs. Data is representative of 2 independent experiments (*n* = 3-5 mice per group). Floating bars show min to max, and line is at mean. CD, control diet; TDD, Trp deficient diet; 5-HT, 5-hydroxytryptamine (serotonin); I3C, indole-3-carbinol; siLPL, small intestinal lamina propria; mLN, mesenteric lymph node.
